# A novel role for nucleolin in splice site selection

**DOI:** 10.1080/15476286.2021.2020455

**Published:** 2022-02-27

**Authors:** Kinneret Shefer, Ayub Boulos, Valer Gotea, Maram Arafat, Yair Ben Chaim, Aya Muharram, Sara Isaac, Amir Eden, Joseph Sperling, Laura Elnitski, Ruth Sperling

**Affiliations:** a Department of Genetics, The Hebrew University of Jerusalem, Jerusalem Israel; b Translational and Functional Genomics Branch, National Human Genome Research Institute, NIH, Bethesda, MD USA; c Department of Natural Sciences, The Open University, Raanana Israel; d Department of Cell and Developmental Biology, The Hebrew University of Jerusalem, Jerusalem Israel; e Department of Organic Chemistry, The Weizmann Institute of Science, Rehovot Israel

**Keywords:** 5ʹ splice site selection, suppression of splicing, alternative splicing, endogenous spliceosome, latent splice sites, latent splicing, splicing regulation, mass spectrometry, RNA sequencing, bioinformatics analysis

## Abstract

Latent 5ʹ splice sites, not normally used, are highly abundant in human introns, but are activated under stress and in cancer, generating thousands of nonsense mRNAs. A previously proposed mechanism to suppress latent splicing was shown to be independent of NMD, with a pivotal role for initiator-tRNA independent of protein translation. To further elucidate this mechanism, we searched for nuclear proteins directly bound to initiator-tRNA. Starting with UV-crosslinking, we identified nucleolin (NCL) interacting directly and specifically with initiator-tRNA in the nucleus, but not in the cytoplasm. Next, we show the association of ini-tRNA and NCL with pre-mRNA. We further show that recovery of suppression of latent splicing by initiator-tRNA complementation is NCL dependent. Finally, upon nucleolin knockdown we show activation of latent splicing in hundreds of coding transcripts having important cellular functions. We thus propose nucleolin, a component of the endogenous spliceosome, through its direct binding to initiator-tRNA and its effect on latent splicing, as the first protein of a nuclear quality control mechanism regulating splice site selection to protect cells from latent splicing that can generate defective mRNAs.

## Introduction

All multi-exon genes undergo regulated splicing, and most are subject to alternative splicing (AS), which provides a major source of diversity in the human proteome and contributes largely to regulation of gene expression (reviewed in refs. [1–6]). Importantly, misregulation of splicing, through mutations or level changes of either regulatory signals or spliceosome components and splicing regulatory proteins, contributes to several human diseases, including cancer [7–10].

A key step in constitutive and alternative pre-mRNA splicing is recognition and selection of a consensus sequence that defines the 5ʹ splice site (5’SS). The 5’SS consensus sequence, AG/GTRAGT (where R denotes purine and ‘/’ denotes the splice junction), is well defined and remarkably similar among many organisms, from mammals to plants [11–13]. Surprisingly, surveys of human gene annotations demonstrated that intronic sequences abound in 5’SS consensus sequences that are not involved in splicing under normal conditions (termed latent 5’SS), exceeding the number of authentic 5’SSs by a factor of 6 to 9-fold [14,15]. Notably, the intronic sequences upstream of almost all (>98%) of these sites harbour at least one in-frame STOP codon, and therefore have the potential to introduce premature termination codons (PTCs) into the alternatively spliced isoforms [15]. mRNAs that contain PTCs are nonsense mRNAs that can be harmful to cells as their truncated proteins are likely non-functional and can have potentially deleterious dominant effects on the cell’s metabolism [16]. Although splicing at latent sites has not been observed under normal growth, latent 5’SSs have been shown to be legitimate and can be activated. Latent splicing has been previously elicited in three different ways: (*i*) by eliminating the STOP codons (in several gene constructs) either by point mutations that converted the STOP codons to sense codons or by indels that shift the reading frame upstream of the STOP codons [17,18]; (*ii*) by disrupting the reading frame through mutating the start ATG codon [19,20]; or (*iii*) by subjecting cells to stress conditions, such as heat shock or in cancer [15,18,19,21,22].

Two scenarios can account for why splicing at latent sites have not been observed under normal growth: (*i*) splicing at latent 5’SSs does occur, but an RNA surveillance mechanism, such as NMD [23–25], rapidly and efficiently degrades the nonsense mRNAs to a level below detection; or (*ii*) there is an unknown suppression mechanism of splicing at latent 5’SSs that are preceded by at least one STOP codon in-frame with the upstream exon. Experiments have ruled out the first scenario of NMD [17–19,22], or degradation by a yet unknown RNA degradation mechanism [20], while fitting the second scenario of latent splicing suppression. Latent splicing was shown to be regulated through the maintenance of an open reading frame [17–20,22] and can be up-regulated under stress conditions [15,21,22]. These discoveries suggest the existence of an RNA quality control mechanism – termed suppression of splicing (SOS) – whose proposed function is to suppress the use of latent 5’SSs that would generate nonsense RNA transcripts [26,27]. Support for a nuclear surveillance mechanism which operates independent of NMD [28] and for nuclear recognition of a PTC-harbouring pre-mRNA and suppression of splicing to prevent such transcripts has been observed in a number of studies [29–31], including a study that showed nuclear retention of unspliced PTC-harbouring transcripts at their genomic loci [32]. However, the mechanism of SOS quality control remains nebulous.

Previous experiments showed that SOS regulation requires an open reading frame [17,18] and an initiation codon [19,20], suggesting a role for the initiator-tRNA (ini-tRNA) in this regulation [20]. Indeed, ini-tRNA, which was found associated with the endogenous spliceosome, was found to act as a pre-mRNA splicing regulator, independent of its role in translation, and was identified as a potential SOS factor [20]. Mutations in the AUG translation initiation codon led to activation of latent splicing, but could be compensated for by expressing ini-tRNA constructs carrying complementary anticodon mutations, which suppressed latent splicing. This effect was specific to ini-tRNA, as elongator-tRNA, whether having complementary mutation to the mutated AUG or not, did not rescue SOS [20]. Thus, ini-tRNA that recognizes the AUG sequence through base pairing is a pivotal element in the predicted SOS mechanism. Its interaction with the AUG sequence, probably in a complex with auxiliary proteins, would establish a register for the recognition of the reading frame required for SOS [20,27,33], which otherwise would not be discernible in the nucleus. Ini-tRNA presumably functions at the initial steps of SOS, conveying SOS components to the pre-mRNA.

Building on the previous discovery that ini-tRNA represents a key element in SOS, here we search for cellular components that directly interact with ini-tRNA in the nucleus to help decipher the SOS mechanism. We identify nucleolin (NCL), an abundant, highly conserved and multifunctional protein, which we previously reported as being associated with the endogenous spliceosome [34], as a new SOS factor. We show that NCL is directly and specifically interacting with ini-tRNA in the nucleus, but not in the cytoplasm. Next, we show association of ini-tRNA and NCL with pre-mRNA. Notably, we show that the recovery of SOS by ini-tRNA complementation is NCL dependent. Furthermore, when we knocked down NCL we observed activation of latent splicing at hundreds of latent 5’SSs that introduce in-frame STOP codons, disrupting gene transcripts involved in several important cellular pathways and cell metabolism functions. These results suggest a novel role for NCL in splice site selection as a component of the SOS quality control mechanism proposed to protect cells from the use of latent 5’SSs that would generate mRNA molecules with PTCs.

## Results

### Search for proteins directly associated with ini-tRNA in the nucleus

**Figure 1.**
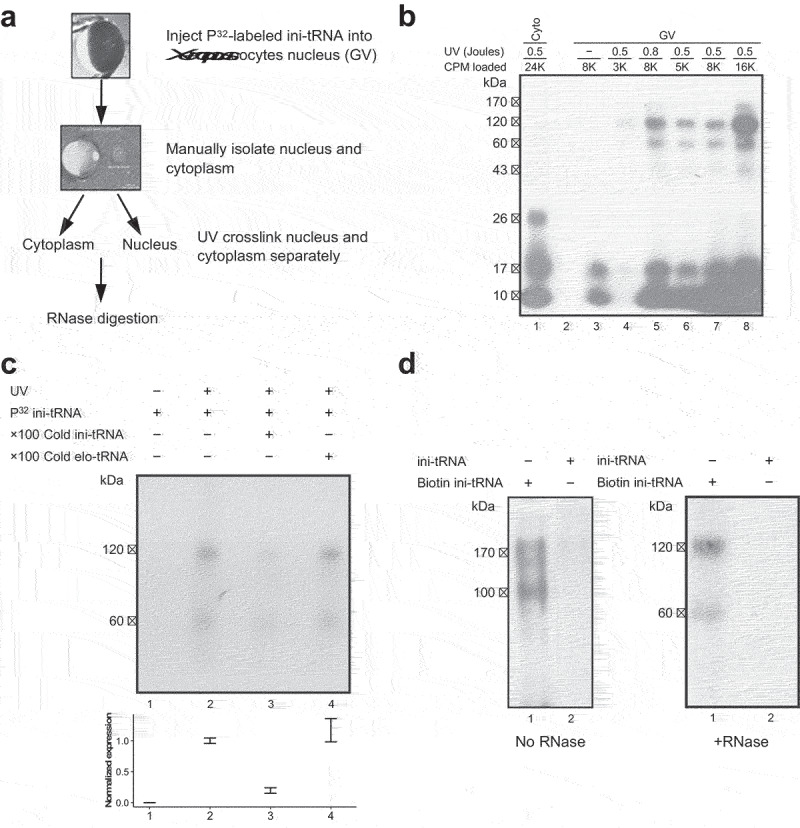
Specific crosslinking of proteins to ini-tRNA in the nucleus. (*A*) Scheme of the experiment. *In vitro* transcribed ^32^P-labelled ini-tRNA was injected into individual nuclei of *Xenopus* oocytes. Isolated nuclei and cytoplasm were crosslinked with UV light, and the crosslinked complexes were further digested with RNase. (*B*) SDS PAGE analysis of aliquots from experiments performed as described in *A*. Lane 1, UV crosslinked cytoplasm; lane 3, non-crosslinked nucleus; lanes 4–8, individual nuclei transfected with the indicated levels of ini-tRNA (cpm) and crosslinked by UV light at the indicated dose. (*C*) Specific association with ini-tRNA. The UV crosslinked bands are chased by 100X cold ini-tRNA, but not by 100X cold elongator-tRNA. Lower panel, quantification of the experiment (4 biological repeats for panels 1–3, and 3 biological repeats for panel 4) (*D*) Affinity purification of proteins bound to ini-tRNA. *In vitro* transcribed biotinylated ^32^P-labelled ini-tRNA was injected into individual nuclei of *Xenopus* oocytes [as described in (A)], UV crosslinked, and affinity purified on streptavidin magnetic beads and run on SDS PAGE. Control, non-biotinylated ^32^P-labelled ini-tRNA.

To further explore the mechanism of SOS, we searched for factors directly associated with ini-tRNA only in the nucleus but not in the cytoplasm. In our first round of experiments, we injected ^32^P-labelled ini-tRNA into either the nucleus or cytoplasm of *Xenopus laevis (Xenopus)* oocytes, followed by UV crosslinking and RNase digestion (Fig. 1A; see Materials and Methods for details). We found ini-tRNA directly associated with specific proteins in the nuclei as represented by two radioactive bands of apparent MW of ~60 and ~120 kDa (Fig. 1B; lanes 5–8). Samples not treated by RNase revealed two major bands of apparent MW of 120 kDa and 170 kDa (see Fig. 1D). The 60 and 120 kDa bands were not found in the cytoplasmic fraction samples (which only contained a ~ 26 kDa band) (Fig. 1B, lane 1) or when the sample was not exposed to UV radiation (Fig. 1B, lane 3).

Importantly, this association of ini-tRNA with the factors represented by the 60 and 120 kDa bands is specific, because these bands, which only appeared after UV crosslinking and were not visible without it (Fig. 1C, lanes 2 and 1, respectively), were chased by the addition of a hundred-fold cold ini-tRNA (Fig. 1C, lane 3). Yet, they were hardly affected by a 100-fold excess cold elongator-tRNA (Fig. 1C, lane 4). It should be pointed out that elongator-tRNA is a suitable negative control, because it is not associated with the endogenous spliceosome and it does not play a role in SOS [20]. Specifically, activation of latent splicing induced by mutations in the translation initiation AUG codon, were suppressed by ini-tRNA constructs carrying anticodon mutations that compensate for the AUG mutations. In contrast, elongator-tRNA, either WT or mutated to complement the mutated AUG did not suppress activation of latent splicing, as demonstrated for three different transcripts [20]. These observations indicate that factors with apparent molecular weight of 60 and 120 kDa were directly and specifically bound to ini-tRNA in the nucleus, but not in the cytoplasm.

To affinity purify the proteins associated with ini-tRNA in the nucleus, the experiment (see Fig. 1A) was repeated, this time by injecting biotinylated ini-tRNA into *Xenopus* oocytes nuclei. Next, biotinylated ini-tRNAs with crosslinked proteins were affinity purified using streptavidin magnetic beads. As can be seen in Fig. 1D, SDS PAGE of affinity-purified ini-tRNA with crosslinked components revealed two bands of associated components with apparent MW of 120 and 170 kDa, which yielded two respective bands of apparent MW of 60 and 120 kDa after RNase digestion (Fig. 1D, lane 1, left and right, respectively). These two bands have the same apparent MW as the bands obtained previously, that is, without affinity purification. The control (affinity-purified non-biotinylated ^32^P-labelled ini-tRNA) did not yield any product (Fig. 1D, lane 2, left and right).

**Figure 2.**
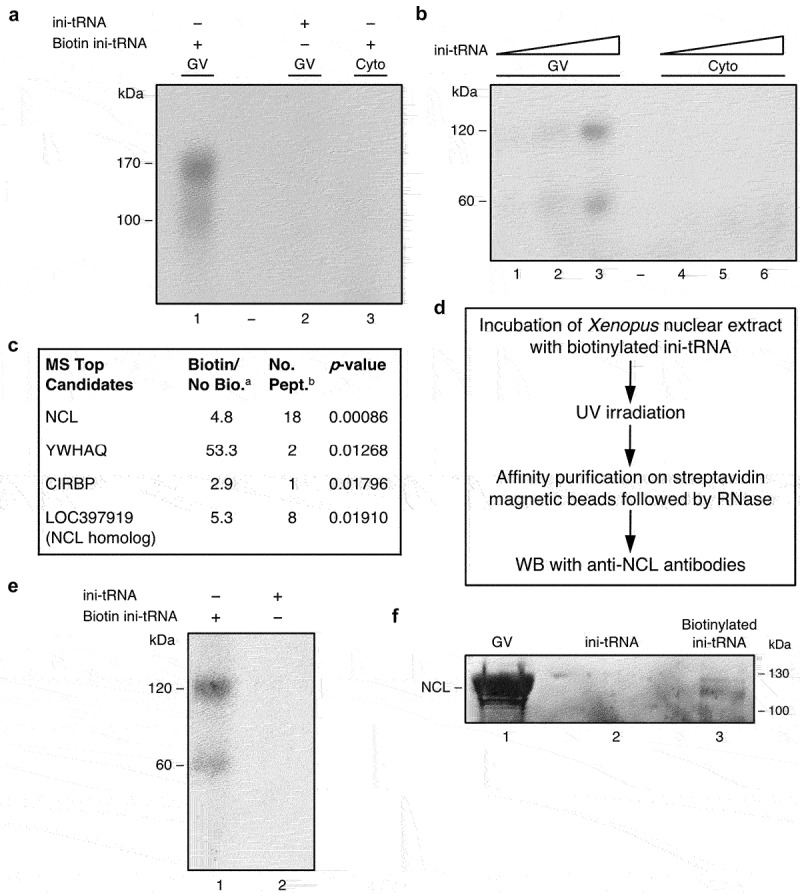
NCL is directly bound to ini-tRNA in the nucleus. (*A, B*) Proteins bound to ini-tRNA in nuclear extracts. Increasing quantities of *in vitro* transcribed biotinylated ^32^P-labelled ini-tRNA were incubated with nuclear (lanes 1–3) and cytoplasmic (lanes 4–6) extracts, UV crosslinked and affinity purified on streptavidin magnetic beads and run on SDS PAGE before (*A*) or after (*B*) RNase treatment. (*C*) Significant peptides associated with ini-tRNA in the nucleus based on mass spectrometry analysis; ^a^Bio/noBio indicates the fold change in biotin versus no biotin, average of three biological experiments; ^b^number of peptides (see also **Table S1**). (*D*) Scheme of the experiment described in *E, F* confirming NCL binding to ini-tRNA. (*E*) *In vitro* transcribed biotinylated ^32^P-labelled ini-tRNA was incubated with nuclear extract of *Xenopus* oocytes, UV crosslinked, and subjected to affinity purification on streptavidin magnetic beads, and run on SDS PAGE after RNase digestion (lane 1), and control non-biotinylated ^32^P-labelled ini-tRNA that went through the same procedure (lane 2). (*F*) Analogous experiment, followed by WB with anti-NCL antibodies. *Left*, GVs; *middle*, control non-biotinylated ini-tRNA; *right*, biotinylated ini-tRNA.

To increase the yield of affinity-purified peptides, we repeated the experiment, this time replacing injection by incubation of biotinylated ^32^P-labelled ini-tRNA with an extract from one isolated *Xenopus* nucleus or cytoplasm. Again, we obtained two bands of apparent MW of 170 and 120 kDa observed only in the nuclear extract and not in the cytoplasmic extract (Fig. 2A, lanes 1 and 3, respectively) nor in the control (Fig. 2A, lane 2). When we incubated our nuclear *Xenopus* extracts with increasing quantities (0.5–2 picomol) of biotinylated ^32^P-labelled ini-tRNA, the RNase treated products yielded 60 and 120 kDa bands again (Fig. 2B, lanes 1–3). The intensity of the obtained bands increased concomitantly with increasing quantities of ini-tRNA. No bands were observed when the experiment was repeated with the cytoplasmic extract (Fig. 2B, lanes 4–6), even with increased levels of ini-tRNA. Thus, the use of nuclear extract in our experiments enabled us to increase the amount of ini-tRNA from 10 fmol/nucleus to 2 picomol/nucleus in the procedure. These experiments show the direct and specific association between ini-tRNA and nuclear components having apparent MW of 60 kDa and 120 kDa.

### Identification of proteins associated with ini-tRNA in the nucleus through mass spectrometry (MS) analysis

Using the successful incubation and affinity purification approach described above, we performed a large-scale experiment, where we incubated nuclear extracts with non-radioactive biotinylated ini-tRNA (experimental samples) and non-biotinylated ini-tRNA (control). We also ran a parallel experiment under the same conditions but with biotinylated ^32^P-labelled ini-tRNA to help identify the relevant bands on the gel. We ran mass spectrometry (MS) analysis on the affinity-purified product (on bead digestion). Fig. 2C summarizes the top results from three such biological repeats. To add stringency to our analysis, we considered genes as positive hits only when the ratio of biotin/no-biotin was larger than 3, with p-value <0.05, and when at least two peptides were identified (for the complete list see **Table S1**). According to the MS analysis, the top candidate protein was nucleolin (NCL). Our MS run identified 18 of its peptides (*p*-value: 0.00086) and also eight peptides from the LOC397919 protein, a homolog of NCL, expressed from the homoeolog chromosome. It should be pointed out that the sequences of eight out of the 18 NCL peptides identified by MS analysis are shared with LOC397919. The remaining 10 peptides have one or two mismatches. All the eight peptides identified directly for LOC397919 have one or two mismatches with NCL sequence, and five of those peptides have one or two mismatches with the peptides we identified directly for NCL. Two additional MS experiments confirmed NCL as the top candidate. In one of these two additional experiments, we extracted the proteins from the 60 kDa and 120 kDa bands and identified NCL in the 120 kDa band. It should be noted that NCL, with its acidic N-terminus, four RNA binding motifs in the middle, and a glycine/arginine-rich C-terminus, undergoes multiple post-translational modifications, such as acetylation, ADP-ribosylation, glycosylation, methylation, and phosphorylation **[**35,36**]**. These modifications affect its migration on SDS gels, giving a band of apparent MW of 120 kDa **[**37**]**. This altered migration is consistent with the migration of the upper band in our experiments (Figs. 1, 2).

### Confirmation of NCL as a candidate protein through western blot

To further validate the association of NCL of apparent MW of 120 kDa with ini-tRNA, we followed our UV crosslinking and affinity purification protocol (see Fig. 2D, E) with Western blot (WB) using anti-NCL antibodies (Fig. 2F). We identified NCL in the 120 kDa band of untreated oocyte nuclei (germinal vesicle [GV] nuclei) (Fig. 2F, lane 1), and in the 120 kDa band of affinity-purified proteins crosslinked to ini-tRNA (Fig. 2F, lane 3), but not in the control (Fig. 2F, lane 2). These results embolden the support for the identification of NCL as a protein that directly and specifically associates with ini-tRNA only in the nucleus of *Xenopus* oocytes.

### Association of NCL and ini-tRNA with pre-mRNA

**Figure 3.**
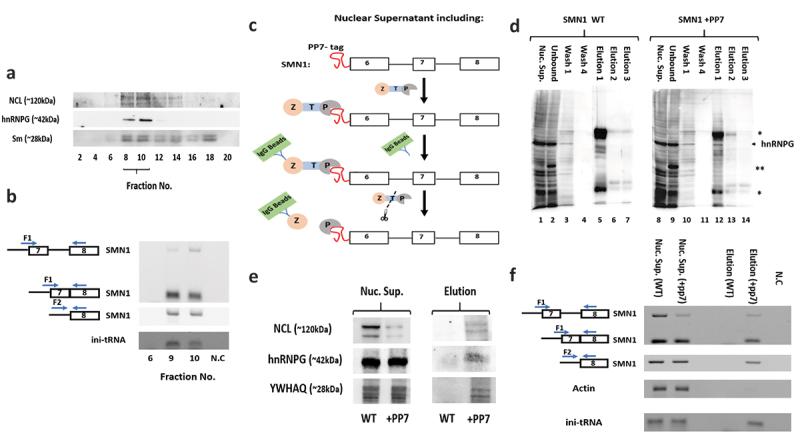
**Ini-tRNA/NCL are bound to pre-mRNA*in vivo***. (A, B) **ini-tRNA/NCL are bound to pre-mRNA in the general population of purified supraspliceosomes**. (A) WB analysis of purified supraspliceosomes. Supraspliceosomes were refractionated on a second glycerol gradient, and aliquots from even fractions were analysed by WB. We use splicing factor hnRNP G to locate 200S supraspliceosomes. Purified supraspliceosomes peak in fractions 8–10. (B) RT-PCR analysis of supraspliceosome peak fractions. Both primers pairs of SMN1 amplify SMN1 pre-mRNA. (C-F) **Affinity purification shows that ini-tRNA/NCL are bound to SMN1 pre-mRNA*in vivo***. (C) Affinity purification scheme. Nuclear supernatants prepared from a HeLa cell line stably expressing the SMN1-PP75ʹUTR transcript, harbouring the PP7 tag at the 5ʹUTR, were incubated with PP7 coat protein bound to protein A via a TEV protease sensitive peptide (ZTP). SMN1 bound components were affinity purified using IgG-coated beads and eluted by TEV protease cleavage. The same protocol was applied to cells stably expressing SMN1 minigene, without the tag, as control. (D) Specific affinity purification of components bound to PP7-tagged SMN1 transcript, WB analysis. Nuclear supernatants prepared from cell lines stably expressing the SMN1-PP75ʹUTR (SMN1+ PP7) transcript (right), or SMN1 WT (SMN1 WT) transcript without the tag (left), were affinity purified. Aliquots from the different steps of the affinity purification were analysed by WB using an anti-hnRNP G antibody. Lanes 1, 8: nuclear supernatant (Nuc. Sup.); lanes 2, 9: material not bound to the beads (Unbound); lanes 3,4, 10,11: washes, 1st and 4th, respectively; lanes 5–7, 12–14: elutions [1–3] (*, heavy and light chains of the IgG antibody used for the affinity purification procedure. **, ZZTEVPP7CP protein). (E) WB analysis of proteins bound to SMN1 pre-mRNA. (F) RT-PCR analysis of bound RNA. Both primer pairs of SMN1 amplify SMN1 pre-mRNA. Actin is used as a negative control. Nuc. Sup., starting material; Elution, bound material; N.C, negative control, PCR of RNA without reverse transcription. Both primers pairs of SMN1 amplify SMN1 pre-mRNA. Identity of bands is given on the left; open boxes, exons; lines, introns; arrows, PCR primers. All DNA bands were confirmed by sequencing.

We have previously demonstrated association of ini-tRNA with the supraspliceosome and interaction of ini-tRNA with pre-mRNA through base pairing, which affects pre-mRNA splicing [20]. We have also shown that NCL is associated with affinity purified specific supraspliceosomes [34]. Here, we showed the formation of a complex of ini-tRNA with NCL (Figures 1, 2). To confirm the interaction of this complex with pre-mRNA, we first purified the general population of supraspliceosomes from HeLa cells. For this aim, supraspliceosomes were fractionated on a glycerol gradient, the supraspliceosome fractions were pooled and refractionated on a second glycerol gradient, as described [38]. Previous studies revealed that this protocol purifies supraspliceosomes of the general nuclear pre-mRNA population, each assembled with spliceosomal U snRNPs and splicing factors in 200S supraspliceosomes, which were also visualized by electron microscopy [38–40]. Here, WB of fractions across the second gradient of purified supraspliceosomes revealed that the splicing factor hnRNP G peaks at the supraspliceosome fractions 8–10, together with NCL and Sm antigens (Fig. 3A). For the analysis of pre-mRNA we have chosen the SMN1 (Survival of Motor Neuron 1) gene transcript. RT-PCR analysis of the supraspliceosome peak fractions revealed that SMN1 pre-mRNA and ini-tRNA are present in the purified supraspliceosome fractions (Fig. 3B), which also contain NCL (Fig. 3A). These studies confirm the presence of NCL, ini-tRNA together with pre-mRNA in the general population of supraspliceosomes.

Next, we analysed affinity purified specific splicing complexes using RNA pulldown. For this aim, we introduced the binding site of *Pseudomonas aeruginosa* Phage 7 (PP7) coat protein at the 5ʹ end of SMN1 minigene (PP7 tag). This system was previously used for affinity purification of native RNP complexes [41] and of specific supraspliceosomes [34]. Our previous studies have shown that splicing complexes assembled in vivo, which were affinity purified using their PP7-tag are assembled in supraspliceosomes and harbour all splicing components, as well as NCL [34]. Here, we used a stable cell line expressing a PP7-tagged SMN1 minigene termed SMN1-PP75ʹUTR minigene, (including exons 6, 7, 8 and IVS 6 and 7), PP7-tagged at the 5ʹ UTR [42]. A stable cell line expressing the SMN1 minigene, lacking the PP7-tag was used as control. Nuclear supernatants enriched in supraspliceosomes were prepared from the stable cell lines expressing SMN1-PP75ʹUTR using our protocol [38,43] (see also Material and Methods). For affinity purification (Fig. 3C), the nuclear supernatants were incubated with the PP7 coat protein fused to two Z domains of protein A by a Tobacco etch virus protease (TEV) cleavage site (ZZTEVPP7CP) [41], and RNA and proteins associated with the SMN1-PP75ʹUTR transcript were affinity purified by first binding to IgG agarose beads, followed by elution using the TEV protease under native conditions, as described [42](See Materials and Methods). The same protocol was applied to nuclear supernatants prepared from the stable cell line expressing untagged SMN1 minigene, as control. WB analysis (Fig. 3D) revealed that the affinity purification was specific, as the splicing factor hnRNP G is found associated only with the PP7-tagged transcript and not in the control. It should be noted that only spliceosomal SMN1 components are expected to be purified by this protocol. RT-PCR analysis also confirm the specific affinity purification of PP7-tagged SMN1 pre-mRNA (Fig. 3F). The specificity of purification is further demonstrated by RT-PCR analysis of actin, which is found only in the nuclear supernatants, but is absent from the eluted affinity purified samples (Fig. 3F). RT-PCR analysis revealed the association of ini-tRNA with the SMN1 pre-mRNA. The association of NCL as well as the splicing factor hnRNP G with the affinity purified SMN1 pre-mRNA is confirmed by WB (Fig. 3E). This association is specific as it is not found in the control sample (Fig. 3F). Notably, the protein YWHAQ ([3–14]-3), identified as associated with ini-tRNA in the nucleus by the UV crosslinking-MS experiments (Fig. 2C) is also specifically associated with the splicing complexes of SMN1 pre-mRNA (Fig. 3E). These studies demonstrate the association of ini-tRNA and NCL in complex with pre-mRNA, in the general population of supraspliceosomes and with a specific pre-mRNA.

### A potential role for NCL in SOS

**Figure 4.**
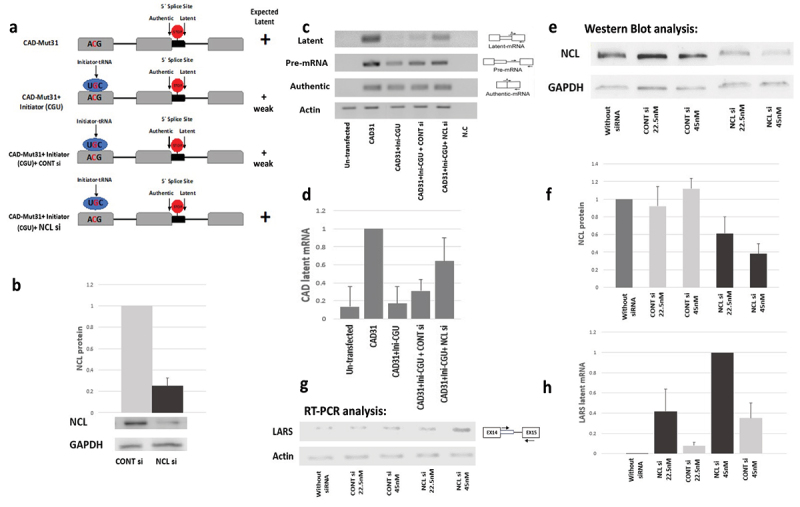
**A potential role for NCL in SOS**. (A-D) **The recovery of SOS by ini-tRNA complementation is NCL dependent**. (A) Hypothesis and experimental design. It was previously shown that abrogation of SOS, caused by mutations in the translation initiation AUG codon can be rescued by expressing ini-tRNA constructs carrying anticodon mutations that complement the AUG mutations [20]. Specifically, it was shown that bypassing SOS in an AUG to ACG mutant (CAD-Mut31), which elicits latent splicing [19], could be rescued by a mutant ini-tRNA that carries a complementary anticodon (CGU) mutation, resulting in a reduced level of latent splicing [20]. We hypothesize that the above rescue of SOS by ini-tRNA complementation is NCL dependent. (B – D) Experimental verification using the CAD minigene. HEK 293 T cells were co-transfected with CAD-Mut31 (CAD31), which carries the mutated ACG start-codon; with mutant ini-tRNA, in which the antisense codon was mutated to CGU (ini-CGU), as indicated; and with si-RNA directed against NCL (NCLsi) and control siRNA control (CONTsi), as indicated. (B) Quantification of NCL knockdown. NCL was analysed by WB and normalized to GAPDH. (C) RT-PCR analysis of: un-transfected cells; cells transfected with: CAD Mut31(CAD31); CAD Mut31(CAD31)+ mutant ini-tRNA (ini-CGU); CAD Mut31(CAD31)+ mutant ini-tRNA (ini-CGU)+control si-RNA (CONTsi); and CAD Mut31(CAD31)+ mutant ini-tRNA (ini-CGU)+siRNA against NCL (NCLsi), using CAD minigene specific primers, as indicated. (D) The block diagrams represent averages of three independent experiments. The densitometric ratio of CAD31 was established as 100%. (E-H) **Decrease in NCL concentration is followed by increase in latent splicing of the endogenous gene transcript LARS**. HEK 293 T cells were transfected with increasing amount of siRNA against NCL (0, 22.5 nM, and 45 nM) and control siRNA. (E, F) Quantification of the NCL knockdown. NCL was analysed by WB (E); and data were normalized to GAPDH, with the level of un-transfected cells taken as 1. (G, H) RT-PCR validation of activation of LSS in intron 14 of the endogenous LARS gene transcript, using the indicated primers. Quantification of the RT-PCR. The densitometric ratio of NCLsi 45 nM was established as 100%. The data are Means ±SD of the independent analyses.

In previous studies, we have shown that abrogation of SOS, caused by mutations in the translation initiation AUG codon can be rescued by expressing ini-tRNA constructs carrying anticodon mutations that complement the AUG mutations. This rescue activity, which depends on codon-anticodon recognition, was shown to be independent of its function in protein biosynthesis. This is because the mutated ini-tRNAs could rescue SOS while inhibiting protein biosynthesis; second, the mutated ini-tRNA appeared to be uncharged with an amino acid. These experiments showed that recognition of the initiation AUG sequence by the anticodon triplet of ini-tRNA plays a role in SOS [20]. Here we used a CAD (Carbamoyl-phosphate synthetase, Aspartate trans-carbamylase, Dihydroorotase) minigene construct (CAD-Mut31) having a latent splice site, and carrying a mutation in the first AUG: AUG-to-ACG [20]. We first repeated the previous experiments (20, see also Fig. 4A), showing that SOS is abrogated when expressing CAD-Mut31, and latent splicing is expressed (Fig. 4C, D). However, overexpression of a mutant ini-tRNA in which the antisense codon was mutated to CGU, to complement the AUG to ACG mutation in CAD pre-mRNA mutant, rescued SOS (Fig. 4C, D). Notably, when NCL is knocked down by siRNA (Fig. 4B) in the same experiment, namely, in a cotransfection experiment of CAD-Mut31 with ini-tRNA Mut CGU that rescue SOS (**see** Fig. 4A), we find that latent splicing is activated, while latent splicing is not activated in control si-RNA (Fig. 4C, D). This experiment reveals that rescue of SOS in CAD minigene mutated in the first AUG by complimentary anticodon mutations in the ini-tRNA is abrogated by knockdown of NCL, showing that SOS function, which is mediated by base pairing of ini-tRNA with the initiation codon of the pre-mRNA, is abrogated by NCL knockdown. This experiment shows the functional requirement of NCL in SOS.

We next analysed how changes in NCL concentration affect latent splicing activation. For this experiment we have chosen to analyse the endogenous LARS (Leucyl-tRNA Synthetase) gene transcript, which has a latent site in intron 14. As shown below (Table S5), knockdown of NCL followed by RNA-seq and bioinformatics analysis revealed LARS as one of the top gene transcripts with activated latent splicing (intron 14). Activation of latent splicing in LARS after NCL knockdown was also validated by RT-PCR (see below). Here, we first show that the use of increasing concentrations of si-RNA directed against NCL is accompanied by gradual decrease in the concentration of NCL as revealed by WB (Fig. 4E, F). RT-PCR analysis of the expression of latent mRNA of the endogenous LARS gene transcript revealed that the decrease in NCL concentration is accompanied by increase in activation of latent splicing in LARS. This experiment shows that lower concentrations of NCL lead to higher usage of the latent splice site.

### Annotating the genomic landscape of latent 5ʹ splice sites

**Figure 5.**
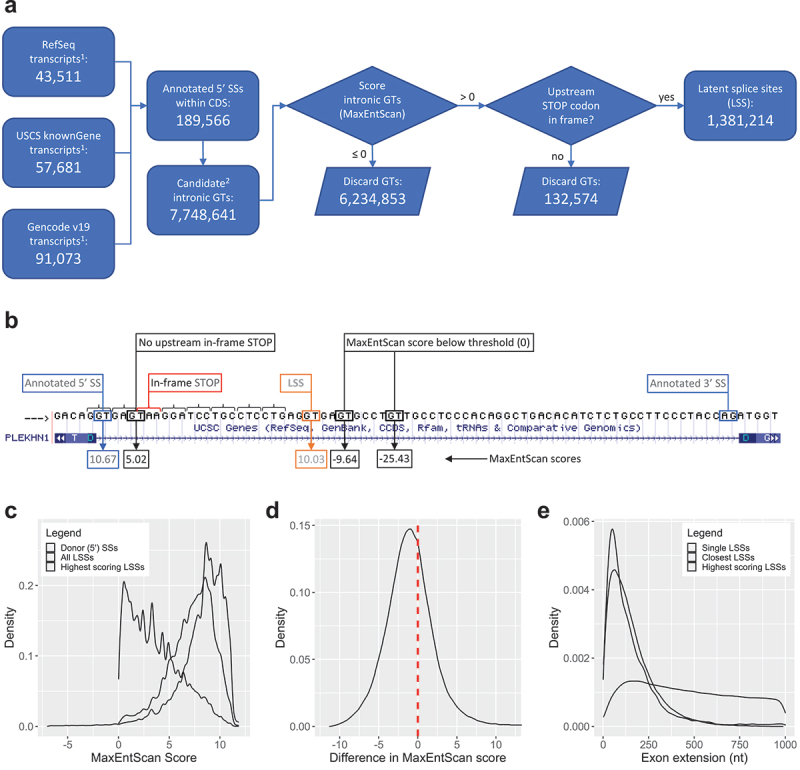
Latent splice sites (LSSs) in human genes. (*A*) Flowchart of computational steps performed to identify LSSs using the hg19 assembly of the human genome. ^1^Number of transcripts reflect only multiple-exon protein-coding transcripts annotated on chromosomes 1–22, X, and Y. ^2^Candidate GTs include only those GTs located within-CDS intronic regions that would extend the upstream exon by at most 1000 nt while preserving a downstream intronic region of at least 20 nt (see also **Table S2**). (*B*) Examples of GTs evaluated for their potential LSS function in intron 15 of the *PLEKHN1* gene (RefSeq accession number NM_032129). (*C*) Distributions of MaxEntScan scores for LSSs and corresponding annotated donor 5’SSs. 0.9% of annotated 5’SSs have scores lower than −5 (not shown), and can be as low as −42.68. (*D*) Distribution of differences in MaxEntScan scores between the highest scoring LSSs located downstream of an annotated 5’SS and the score of that 5’SS. 34.2% of 5’SSs have at least one stronger LSS downstream of them. (*E*) Distributions of distances between LSSs and corresponding 5’SSs (i.e. exon extensions). ‘Single LSSs’ denotes those cases where a single LSS can be found downstream of a specific 5’SS (8886 cases). For 5’SSs with more than one LSS downstream (159,931 cases), distributions of exon extensions for both the closest and strongest LSSs are shown.

To assess whether NCL is affecting regulation of splicing at latent 5ʹ splice sites (here termed LSS), we first compiled a comprehensive annotation of these sites in the genome. We previously used a bioinformatics approach to identify potential LSSs in intronic sequences across the human genome, using data generated from expression exon microarrays [15]. Using stringent criteria, we showed that LSSs are present in most human introns represented on the array [15]. Here, we used a more inclusive scoring threshold (MaxEntScan score [44] of 0 for intronic GT dinucleotides) to compile a comprehensive set of potential LSSs, filtered by known splice sites from multiple human gene annotation databases (see Materials and Methods for explanation, Fig. 5A,B). This score threshold was chosen because it excluded the majority of intronic GT dinucleotides (less than 20% of intronic GTs score higher than 0) but was inclusive of potential new LSSs because more than 98.5% of annotated 5’SSs score higher than 0 (**Figure S1**). Limiting our search to LSSs located <1 kb downstream of annotated 5’SSs, we found a total of 1,381,214 LSSs (see Materials and Methods; **Table S2**). The majority of protein coding genes (nearly 89%) contained at least one LSS, with an average of nearly 80 LSSs per gene (median value: 55). The genes with the highest number of LSSs were *NBPF14* and *NOTCH2NLB*, which contained 1,190 LSSs each, whereas 72 other genes contained only one LSS. In general, we observed between 1 and 93 LSSs (median count: 8) within 1kb downstream of annotated 5’SSs, with multiple LSS occurrences being typical (in nearly 95% of cases).

Most LSSs had lower MaxEntScan scores than the annotated 5’SSs upstream of them (median values of 3.19 and 8.68, respectively), and their score distribution was strongly skewed towards lower values (Fig. 5C). However, if only the highest scoring LSSs downstream of each 5’SS were considered, the MaxEntScan score distribution was similar to the distribution of scores of annotated 5’SSs, albeit slightly shifted towards lower values (Fig. 5C), highlighting the functional potential of intronic GT dinucleotides. For more than one-third of established 5’SSs, there was at least one LSS downstream that scored higher (Fig. 5D), highlighting the importance of the SOS mechanism for suppressing competition from these LSSs. The use of the highest scoring LSS instead of the annotated 5’SSs extended the upstream exon by an average of 469 bp (Fig. 5E) and incorporated an in-frame STOP codon. In all but 15.5% of cases with multiple LSS occurrences, the highest scoring LSS was preceded by LSSs with weaker MaxEntScan scores.

### NCL knockdown by siRNA and RNA-seq

**Figure 6.**
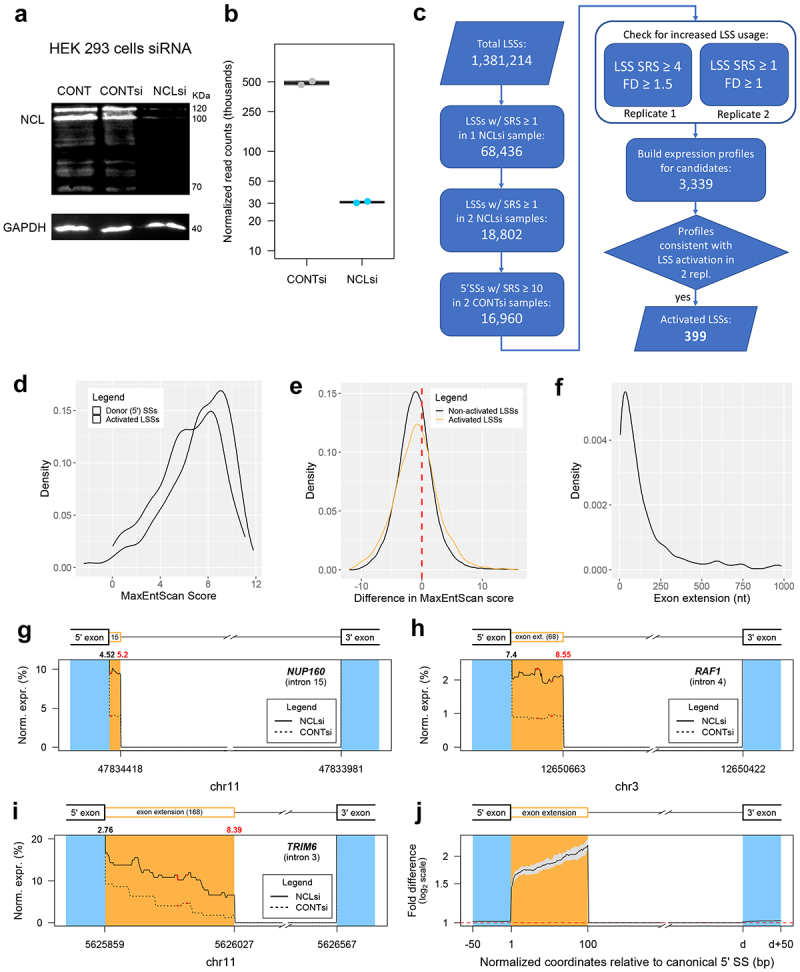
Identification of LSSs activated by knockdown of NCL. (*A, B*) HEK 293 cells were treated by siRNA against NCL (see Materials and Methods). As controls, we used non-targeting siRNA #1(Dharmacon), and non-treated cells. Proteins and RNA were extracted after 48 hr. (*A*) Proteins were analysed by SDS PAGE. (*B*) Analysis of NCL expression using RNA-seq data (see Materials and Methods). Normalized reads of RNA from cells treated by siNCL (NCLsi) and siControl (CONTsi) (biological duplicates from each) are presented (see also **Table S4**). (*C*) The computational pipeline for identification of activated LSSs. Selection of LSS activated candidates was done based on the presence of split reads supporting the LSS junction. Replicate 1 and 2 can refer to either sample replicate pairs. Expression profiles for all candidates were analysed individually to assure consistency between the two replicates. SRS – split read support (see also **Table S5**). (*D*) Distribution of MaxEntScan scores for the 399 activated LSS and the corresponding 385 annotated 5’SS upstream of them. (*E*) Distribution of differences in MaxEntScan scores between the activated LSSs and the corresponding upstream 5’SS (score_5’SS_ – score_LSS_). There are 401 unique LSS-5’SS pairs. Data for the set of non-activated LSSs was obtained with the highest scoring LSSs downstream of the 103,375 5’SSs with split read support of at least 10 reads for the canonical junction in both CONTsi samples. (*F*) Distribution of lengths for exon extensions caused by LSS activation. (*G-I*) Typical expression profiles of exon extension regions upstream of LSSs activated in NCLsi samples. Positions of in-frame STOPs are shown by red segments. Numbers on top of the graph indicate MxEntScan scores for the annotated 5’SS (black) and the LSS (red). The length of the exon extension is indicated in the corresponding segment of the gene model. (*J*) Composite profile showing the fold difference in expression level between NCLsi and CONTsi samples for all 399 cases of activated LSSs. The black line represents median fold difference values at nucleotide resolution, whereas the grey area represents the 95% confidence interval for the median.

To further explore the role of NCL in SOS, we knocked down NCL in HEK 293 cells using siRNA in two biological replicate samples (referred to as NCLsi samples), from which we isolated proteins and RNA (see Materials and Methods). Our controls were from two paired biological replicate samples treated with non-targeting siRNA (referred to as CONTsi), and untreated cells (referred to as CONT). Western blot analysis indicated that the NCL protein level was knocked down 25-fold (Fig. 6A). HiSeq RNA sequencing (RNA-seq) analyses yielded more than 230 million reads (126-bp, paired-end) for each sample, and about 84% of them were uniquely aligned to the hg19 assembly (**Table S3**). Standard differential expression analysis by DESeq2 revealed that NCL was the gene with the highest decrease in expression and the greatest fold change, as expected (**Table S4**). The normalized read counts for the NCL locus were reduced by more than 15-fold upon NCL knockdown (see Fig. 6B; the DESeq2 model-based estimate of NCL downregulation is nearly 10-fold, FDR adjusted p = 3.5 × 10^−44^). Therefore, the WB and RNA-seq analyses consistently indicated successful knockdown of NCL.

To test if the NCL knockdown by siRNA has any effect on the cell cycle, we performed flow cytometry analyses after NCL knockdown under the same conditions as for the RNA-seq analysis. HEK 293 cells were treated with siRNA against NCL, in three biological replicates. Untreated cells and cells transfected with the non-targeting siRNA, as above, were used as control. We also repeated the same experiments using lower concentration of siRNA. The results of this experiment revealed that no significant changes in cell cycle were found after knockdown of NCL for 48 hr, compared to untreated or CONTsi transfected cells. **(Figure S2).**

### NCL knockdown activates latent splicing

**Figure 7.**
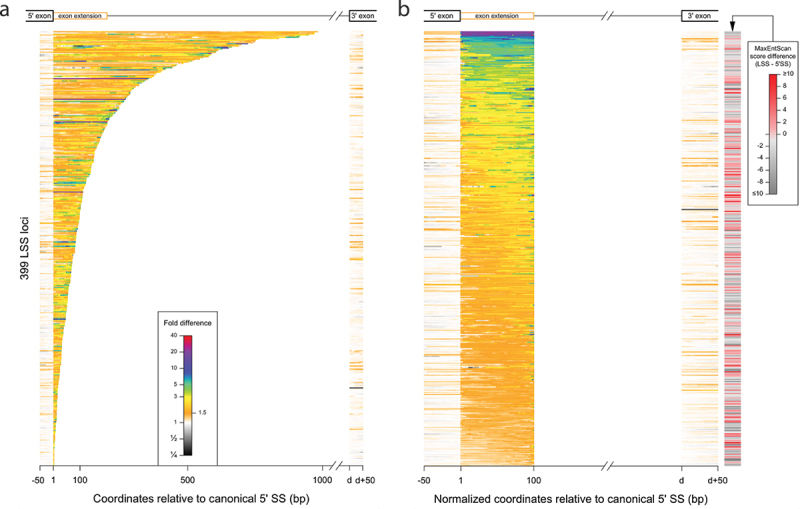
Overview of LSS activation upon NCL knockdown. Colour-coded levels of fold-difference in expression levels is shown for all 399 activated LSSs. Fold difference was computed using normalized expression levels, where the normalization factor was the number of split reads supporting the corresponding annotated 5’SS (see also **Table S5**). (*A*) Data are shown for the actual length of exon extension regions, and LSSs are sorted based on the exon extension length. (*B*) LSSs are sorted based on the average fold difference across the exon extension region, which is shown as a standardized length of 100 bp. The difference in MaxEntScan score between the LSS and the corresponding 5’SS is shown on the right. Shades of red correspond to cases where the LSS has a score higher or close to the score of the corresponding 5’SS. The score is not significantly correlated with the increase in expression observed for the exon extension (Spearman’s *ρ* = −0.051, p = 0.3).

A detailed evaluation of the effect of NCL knockdown on splicing at LSSs was enabled by the depth of RNA sequencing. We used split reads (i.e. reads that align over two consecutive exons through an intron-induced gap) as the basic measure for LSS usage. These reads connect LSSs with the downstream acceptor SSs and represent direct evidence for the splicing of the isoform of interest. We found more than 35,000 LSSs in each sample being supported by at least one split read (1-NCLsi: 43,679; 2-CONTsi: 35,865; 7-NCLsi: 44,124; 8-CONTsi: 41,576). Overall, nearly 5% of all LSSs are supported by at least one split read in at least one of the NCLsi samples (Fig. 6C). After adjusting for differences in the total number of mapped reads for each sample, we found in each replicate an excess of >4% (i.e. more than 1700) in the number of LSSs supported by split reads in NCLsi compared with CONTsi, supporting a role for NCL in regulating splicing at LSSs. To verify that NCL knockdown is linked to activation of splicing at many LSSs, we established a computational pipeline (Fig. 6C) to separate cases of *bona fide* LSS activation from cases that are technical (e.g. spurious alignments) or biological (e.g. unannotated exons) artefacts. To define activated LSS, we required the NCLsi samples to exhibit increased usage of the LSS relative to the annotated upstream 5’SS, as well as higher levels of mapped reads throughout the exon extension region when compared to CONTsi samples (see Materials and Methods). We identified 399 LSSs in 362 genes that conform to all selection criteria (**Table S5**). The MaxEntScan score distribution for these 399 activated LSSs mimicked the score distribution for the 385 corresponding 5’SSs, with a slight shift towards smaller values (Fig. 6D). Notably, 38.7% of activated LSSs showed higher or equal MaxEntScan scores to the corresponding upstream 5’SS (Figs. 6E**, 7B**). This finding, together with the similarity of the score distribution of activated LSSs to that of annotated 5’SSs, indicates that activated LSSs resemble genuine, annotated 5’SSs. Activated LSSs lead to exon extensions that range between 4 and 983 nt, with a median value of 81 nt (Fig. 6F). Three cases of activated LSSs are illustrated in Fig. 6G**-I**, and a median profile of the increased expression throughout the exon extension region for all 399 cases of activated LSSs is shown in Fig. 6J.

The heatmaps (Fig. 7) provide a comprehensive view of the increase in expression associated with all activated LSSs upon NCL knockdown, illustrating the wide range of length distribution of activated latent exons (Fig. 7A). Fig. 7B displays the average fold difference of activation of latent exons upon NCL knockdown, each standardized to a length of 100 nt. It also portrays the MaxEntScan score difference between the activated LSS and the corresponding upstream 5’SS. Four activated LSSs are located in the *NCL* gene itself: three in intron 9 and one in intron 10. In Fig. 7B, these four LSSs appear at the top of the graph, as they show the highest increase in expression levels of the exon extension regions.

We noted that LSS activation can be detected in isoforms with a wide range of expression levels. In a small number of cases, we observed increased usage (i.e. activation) of the same LSS within the context of different transcripts (i.e. the LSS is spliced with two different downstream 3’SSs, or with two different upstream 5’SSs). For example, an LSS located in intron 4 of *HMGN1* (chr21:40,719,840) appeared activated in two isoforms whose expression levels differ by almost 30-fold between the isoforms.

### Gene transcripts affected by NCL knockdown

**Figure 8.**
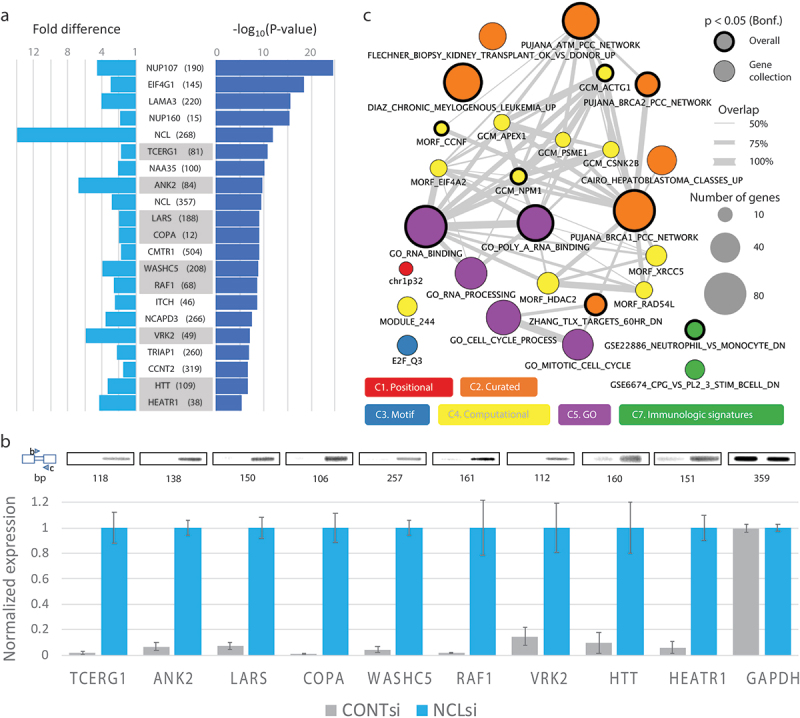
Genes with activated LSSs upon NCL knockdown. (*A*) List of 20 activated LSSs with strongest support based on split reads from the RNA-seq experiments. Cases are ranked based on the p-value obtained using one-sided Fisher’s exact test with the number of reads supporting the LSS and the annotated 5’SS in the NCLsi and CTRLsi samples (values of the two replicates were combined using Fisher’s method). The fold difference in normalized LSS usage between NCLsi and CTRLsi samples is shown in light blue (the value shown corresponds to the geometric mean between the two replicates). Name of the gene and length of the exon extension are also provided (complete details for these cases can be found in **Table S5**). Cases experimentally validated through RT-PCR are highlighted. The case in the *HEATR1* gene was ranked 32nd, but is included here to show it was also experimentally validated. (*B*) RT-PCR validation of activation of latent splicing at LSSs in nine genes expressed in NCLsi treated HEK 293 T cells. CONTsi treated cells were used as the control. Numbers below PCR bands represent the sizes of the PCR products obtained with primers designed to match the schematic representation shown on the left (boxes represent exons, narrow box represents latent exon, i.e. exon extension, triangles represent primers). Bars represent averages and SEMs for three biological replicates. *TCERG1*, Transcription Elongation Regulator 1; *ANK2*, Ankyrin 2; *LARS*, Leucyl-tRNA Synthetase; *COPA*, Coatomer Protein Complex Subunit Alpha; *WASHC5*, WASH complex subunit 5; *RAF1*, Proto-Oncogene, Serine/Threonine Kinase; *VRK2*, vaccinia-related kinase (VRK) of serine/threonine kinase 2; *HTT*, Huntingtin; *HEATR1*, HEAT Repeat Containing 1. GAPDH, glyceraldehyde-3-phosphate dehydrogenase was used for normalization. All PCR products were verified by sequencing. (*C*) Gene sets from MSigDB over-represented among the 362 genes with activated LSSs. Circle areas correspond to the number of genes. Gray lines connect gene sets that share at least 50% of the genes in the smaller set. Thick circle borders correspond to gene sets that remain significant after stringent overall Bonferroni correction for multiple testing (17,810 total tests) (see also **Table S6**).

The cases with the strongest split read support for LSS activation after NCL knockdown are illustrated in Fig. 8A (see also **Table S5**). This list includes proteins involved in several important cellular pathways and cell metabolism functions, such as transcription factors, oncogenes, kinases, splicing factors, translation factors, genes affecting cell motility, proliferation, and cellular trafficking, as well as NCL. These important functions can be found associated with many of the 362 genes affected by LSS activation, in agreement with the SOS being a general quality control mechanism.

For validation of the RNA-seq results through an independent method, we selected nine gene transcripts (*ANK2, TCERG1, LARS, WASHC5, COPA, RAF1, VRK2, HEATR1*, and *HTT*) that are among the cases with the strongest RNA-seq evidence for LSS activation (See Fig. 8A). Using RT-PCR, we validated the RNA-seq results showing that latent splicing was significantly elevated in response to knockdown of NCL as compared with the control cells (Fig. 8B).

To learn whether LSS activation affects specific gene categories preferentially, we evaluated the over-representation of genes with activated LSSs among the gene sets included in all eight gene collections that make up the MSigDB database (i.e. numerous gene sets are within each collection).

We found 27 gene sets with significant enrichment (after Bonferroni correction at gene collection level), 11 of which remained significant after stringent Bonferroni adjustment for the total number of 17,810 gene sets in MSigDB (Fig. 8C**, Table S6**). The most significant (p = 5.9 × 10^−10^) and largest overlap (84 genes) observed was for genes that encode RNA binding proteins according to their gene ontology annotation. Subsets of these genes make up large fractions of many of the other 27 gene sets with significant enrichments (**Table S6**), which could explain the diversity of gene sets significantly affected by LSS activation. Moreover, given that many RNA binding proteins have essential cellular roles that include repair of DNA damage, DNA replication, chromatin regulation, transcription, splicing, translation, protein folding, and cell proliferation, these results suggest that the SOS mechanism plays a critical role in protecting the integrity of cellular functions.

### Further evidence supporting the relevance of NCL in SOS

Our transcriptome survey through RNA-seq revealed increased latent splicing in hundreds of coding transcripts upon NCL knockdown. However, activation of latent splicing can be confounded and exacerbated by experimental stress [15,26], such as the transfection procedure itself. To address this possibility, we switched sample labels between cases and controls and re-applied the computational pipeline to identify LSSs with increased usage. Under these conditions, we identified only 82 instances of LSSs activated in CONTsi compared to NCLsi samples (Table 1, **S7**), nearly 5-fold fewer than identified with original sample labels. This finding indicates that NCL knockdown, rather than experimental stress, represents the main driver for the increase in latent splicing observed in NCLsi compared to control samples.

**Table 1.** NCL knockdown preferentially activates splicing at latent 5’SS that introduce PTCs

A5SS^a^ types	Introduce PTC	A5SS activated per sample type, initial criteria^b^			A5SS activated per sample type, stringent criteria^c^		
		NCLsi	CONTsi^d^	p-value^e^	NCLsi	CONTsi^d^	p-value^e^
LSS	Yes	399	82	2.3 × 10^−11^	187	23	4.2 × 10^−5^
adSS_3n_	No	50	50	N/A	25	16	N/A
adSS_fs_	Yes	221	52	8.5 × 10^−9^	112	20	1.6 × 10^−3^

^a^Alternative 5ʹ splice site. ^b^Initial activation criteria require read support of at least 4 for the latent junction and increase in relative usage of at least 50% relative to the control sample in one replicate. For the second replicate, the latent junction is required to be supported by at least one read, while any increase in the relative usage is observed. ^c^Activation criteria (1.5-fold increase, read support of at least 4) are imposed to both replicates. ^d^Activation of a 5SSs is determined by switching NCLsi and CONTsi sample labels and applying the pipeline described in Fig. 5C.

^e^P-value was computed with one-sided Fisher’s exact test against the adSS_3n_ set.

To investigate whether the role of NCL is specifically linked to the presence of PTCs, we analysed cases of latent splicing where no PTCs are incorporated. Specifically, these cases have no in-frame STOP codon in the exon extension region, and the length of the exon extension is a multiple of three, so as not to change the reading frame. We identified a total of 43,702 such potential alternative donor SSs, referred to as adSS_3n_ (**Table S8**). Using the methodology described in Fig. 6C for LSSs, we identified 50 cases of activated adSS_3n_ in the NCLsi samples compared to controls (Table 1, **S9**). Upon label switching, we also identified 50 instances (**Table S10**), suggesting that NCL knockdown has little impact on the choice of alternative donor SSs when no PTCs are introduced. Indeed, comparison of the LSS and adSS_3n_ activation reveals a significant bias towards LSS activation in NCLsi samples, that is, upon NCL knockdown (Table 1). The same effect was observed even with more stringent criteria for activation of latent splicing (Table 1), supporting the hypothesis of NCL involvement in a mechanism that prevents generation of PTC-containing mRNAs.

To further investigate the relationship between NCL knockdown and the presence of PTCs, we also analysed cases of latent splicing where PTCs are incorporated in downstream exons through a shift of the reading frame. For this purpose we searched for alternative donor SSs that do not introduce PTCs in the exon extension, but extend the exon by lengths that are not multiple of three, which would consequently cause a reading frame shift and insertion of a PTC downstream. We found a total of 98,349 such potential alternative donor SSs, referred to as adSS_fs_ (**Table S11**). Analysis of RNA-seq data revealed 221 cases of activated adSS_fs_ in NCLsi compared to control samples (Table 1**, S12**), and 52 activated adSS_fs_ sites were found upon label switching (Table 1, **S13**). No significant difference can be observed between activation patterns of LSSs and adSS_fs_ (p = 0.28, initial criteria; p = 0.17, stringent criteria, one-sided Fisher’s exact test), supporting a general role for NCL in suppressing PTC-inducing splicing events. In contrast, these data reveal a significant bias towards activation of adSS_fs_ compared to adSS_3n_ in NCLsi samples (i.e. upon NCL knockdown), similar to LSSs (Table 1). Therefore, our multiple complementary analyses support a general role of NCL in preventing PTC-harbouring mRNAs.

## Discussion

### NCL is directly associated with ini-tRNA in the nucleus

Previous studies showed that ini-tRNA, which is associated with the endogenous spliceosome, plays a role in SOS [20]. Here, we identified NCL as directly and specifically associated with ini-tRNA in the nucleus, and not in the cytoplasm. Through our UV crosslinking experiments, we identified factors that interact directly and specifically with ini-tRNA in the nucleus. The specificity of interaction of ini-tRNA with the bound factors was demonstrated by chase experiments using a hundred-fold excess of cold ini-tRNA. However, elongator-tRNA, which does not play a role in SOS, and is not associated with the endogenous spliceosome [20], when used at the same concentration (100-fold excess of cold elongator-tRNA) did not affect the interaction of ini-tRNA with the bound factors (Fig. 1C, lanes 3 and 4, respectively). Affinity purification and mass spectrometry analyses of the crosslinked components revealed NCL as a protein directly and specifically associated with ini-tRNA in the nucleus but not in the cytoplasm. The association is specific as demonstrated by the competition experiments. Furthermore, it should be pointed out that although NCL is an abundant protein, the level of NCL protein expression in *Xenopus* oocytes stage IV–V, used here, is low [45,46]. The identification of NCL as bound to ini-tRNA is in agreement with earlier findings of the association of NCL with the endogenous spliceosome [34].

### NCL and ini-tRNA are associated with pre-mRNA

The finding of NCL and ini-tRNA with pre-mRNA is demonstrated here first by analysing the general population of purified supraspliceosomes (Fig. 3A, B). Previous studies showed that supraspliceosomes represent the general nuclear pre-mRNA population, each assembled with spliceosomal U snRNPs and splicing factors [38,47]. Here, we also analysed the presence of NCL and ini-tRNA with SMN1 pre-mRNA. We used affinity purified PP7-tagged splicing complexes assembled *in vivo* on transcripts expressed from cells stably transfected with SMN1 minigene. We demonstrate the association of ini-tRNA and NCL with the pre-mRNA of SMN1 (Fig. 3C-F). These findings indicate a role for NCL in the spliceosome, which we extend to further define a role in the SOS mechanism.

### Recovery of SOS by ini-tRNA complementation is NCL dependent

Previously, we have shown that SOS is abrogated by mutations in the translation initiation AUG codon. The abrogation of SOS was attributed to mutation in the AUG sequence, rather than interference with splicing control elements, because mutating nucleotides in the vicinity of the AUG sequence did not elicit latent splicing [19]. Notably, this abrogation can be rescued by expressing ini-tRNA constructs carrying anticodon mutations that complement the AUG mutations. This rescue activity of ini-tRNA in splicing, which depends on codon-anticodon recognition, was shown to be independent of its function in protein biosynthesis. This is because the mutated ini-tRNAs could rescue SOS while inhibiting protein biosynthesis; second, the mutated ini-tRNA appeared to be uncharged with an amino acid. These experiments showed that recognition of the initiation AUG sequence by the anticodon triplet of ini-tRNA plays a role in SOS [20]. Here we repeated this experiment, first, by showing that CAD-Mut31, in which the AUG was mutated to ACG, elicits latent splicing. This use of latent splice site can be suppressed by a mutated ini-tRNA harbouring a mutation that complements the mutated AUG (Fig. 4). Notably, we show that SOS function, which is mediated by base-pairing of ini-tRNA with the initiation sequence of the pre-mRNA, is abrogated upon NCL knockdown (Fig. 4A-D). This experiment shows the functional requirement of NCL in SOS.

A further link between NCL and SOS is shown in the experiment following latent splicing after decrease in concentration of NCL, and showing increase in latent splicing in the endogenous LARS gene transcript (Fig. 4 **E-H**).

### NCL – a multifunctional protein

The results of our current study show that NCL has a previously undescribed role in splice site selection. NCL, an RNA binding protein with four RNA binding domains, is known to perform multiple functions in numerous cell locations (nucleolus, cytoplasm, cell membrane and nucleoplasm) and to incur multiple post-translational modifications that affect its cellular location and function [35,48]. It is involved in the synthesis and maturation of ribosomes in the nucleolus, and while many of its functions in other cell components are described [e.g. a role in Pol II transcription, DNA repair, chromatin decondensation, and genome stability [35,49,50]], most of the molecular details of its assorted functions are not understood. Furthermore, NCL is known to play a role in cancer, where its overexpression affects cell survival, proliferation, and invasion [35,50,51]. Our characterization of a previously unknown function for NCL is an important addition to the current body of knowledge of a protein crucial to cell function and involved in an important and widespread disease, cancer.

Recent studies have indicated a connection of NCL with splicing factors [52–56]. Previously, we reported NCL as an integral component of the endogenous spliceosome (supraspliceosome), where it is associated with supraspliceosomes assembled on specific transcripts at all splicing stages [34]. Furthermore, NCL was also identified in association with the general population of supraspliceosomes [57]. While our previously published reports did not define a specific role for NCL in splicing regulation, as we do here, all our findings support a novel role for NCL in splicing regulation.

### NCL proposed as a novel regulator of splice site selection to protect cells from insertion of PTC

Our RNA-seq analysis revealed activation of 399 LSSs when NCL was downregulated. It should be pointed out that the number of activated LSSs uncovered here is likely a conservative estimate, because all the latent mRNAs are PTC-bearing and would likely become substrates for downregulation by NMD, as was previously demonstrated [15], limiting their detection by RNA-seq. Notably, we were able to further validate a subset of 9/9 of these cases by RT-PCR (*TCERG1*, Transcription Elongation Regulator 1; *ANK2*, Ankyrin 2; *LARS*, Leucyl-tRNA Synthetase; *COPA*, Coatomer Protein Complex Subunit Alpha; *WASHC5*, WASH complex subunit 5; *RAF1*, Proto-Oncogene, Serine/Threonine Kinase; *VRK2*, vaccinia-related kinase (VRK) of serine/threonine kinase 2; *HTT*, Huntingtin; *HEATR1*, HEAT Repeat Containing 1). This validation provides support for our bioinformatic approach. Importantly, the MaxEntScan score distribution of activated LSSs closely resembles the distribution of genuine, annotated 5’SSs (Fig. 6D). Furthermore, 38.7% of activated LSSs show higher or equal MaxEntScan scores to the corresponding upstream 5’SS (Figs. 6E**, 7B**). For annotating potential LSSs we have used a rather relaxed MaxEntScan that covers 98.5% of 5’SSs. Notably, a large fraction of activated LSSs can be detected even when more stringent MaxEntScan score thresholds are imposed for LSS selection. For example, a very stringent score threshold of 6.77, encompassing 80% of annotated 5’SSs, allows us to detect 191 activated LSSs (48% of the activated LSSs). Even with this stringent criterion, we detect highly significant bias towards LSS activation compared to adSS_3n_ (p = 7.2 × 10^−7^, one-sided Fisher’s exact test) upon NCL knockdown.

As discussed above, NCL is a multifunctional protein affecting several cellular processes. A number of studies have analysed the effect of NCL knockdown on specific cell functions [56,58–66]. For example, knockdown of NCL was shown to decrease cell proliferation and induce cell cycle arrest, which can cause increased apoptosis [58,59]. Knockdown can also affect the epidermal growth factor (EGF) pathway by decreasing the level of essential factors like p-ERK1/2 and p-AKT [60] and reducing the concentration of 45S rRNA [58]. In chicken DT40 cells, knockdown of NCL inhibited rDNA transcription and cell proliferation, with only a slight effect on pre-rRNA processing, leading to the conclusion that cell division and survival, but not sequence-specific RNA binding, define NCL function [65]. However, despite the breadth of NCL activity, not all processes in the cell are affected by NCL knockdown. For example, the amount of fibrillarin and B23 protein, known to be involved in diverse cellular processes, such as ribosome biogenesis, centrosome duplication and cell proliferation, are not affected by the decrease of NCL level [58]. NCL also modulates the activities of several DNA repair enzymes [61,62]. Knockdown of NCL inhibited the phosphorylation of DNA-dependent protein kinase (DNA-PKcs), which is vital for its role in DNA damage repair and radiation sensitivity [61]. In addition, DNA damage response (DDR) proteins, PCNA and H2AX, interact with NCL during replication stress [62]. Here again, NCL did not affect all proteins involved in DDR, such KU70, a factor of DNA non-homologous end joining, whose expression did not change upon knockdown of NCL [62]. The majority of nuclear proteins interacting with NCL are ribosomal, rRNA processing, and ribosome biogenesis proteins, yet, a third of the interacting proteins were involved in pre-mRNA metabolism, including a number of splicing factors. Notably, knockdown of NCL did not affect the quantity and localization of tested interacting splicing and export proteins [56]. The latter finding is in support of our proposal that the changes in latent splicing that we find upon NCL knockdown are due to changes in NCL concentration and not due to changes in other splicing factors. Beyond RNA processing, a global analysis of the effect of NCL knockdown on mRNA and microRNA expression in HeLa cells, using microarray, showed down regulation of enzymes involved in cholesterol biosynthesis and fatty acid degradation [67].

It should be noted that the studies of the effect of NCL knockdown on cell functions, discussed above, evaluated knockdown effects after several days. However, unlike the above studies, we assessed the global impact of NCL knockdown at 48 hours. It should be pointed out that at this time point no significant changes in the cell cycle were found (Figure S2). Furthermore, at this time point the number of genes with significant (FDR<0.1) differential expression is relatively low (75 gene transcripts) (**Table S4**), compared to analysis of NCL knockdown impact after five days by Kumar et al. (2017), who identified hundreds of affected gene transcripts. For example, we did not find any gene with cell cycle and DNA repair functions among the 75 genes identified with significant differential expression in cases compared to controls (**Table S4**), while Kumar et al. (2017) found tens of genes with cell cycle-related functions. We can thus conclude that the previous studies of the effect of NCL knockdown on cell functions, discussed above, should not affect our proposal for a role for NCL in splicing.

We have shown here that NCL is directly bound to ini-tRNA in the nucleus, and that NCL and ini-tRNA are associated with pre-mRNA. Furthermore, we show that the recovery of SOS by ini-tRNA complementation is NCL dependent, revealing the functional requirement of NCL in SOS. These findings together with the activation of 399 LSSs when NCL was downregulated strongly support our proposal for the involvement of NCL in a mechanism that selects splice sites and thereby prevents insertion of PTCs into mature transcripts.

The gene transcripts (see Fig. 8A and **Table S5**) that underwent activation of latent splicing when NCL was downregulated encode for proteins that are involved in several important cellular pathways and cell metabolism functions; they include transcription factors, oncogenes, kinases, splicing factors, translation factors, and genes affecting cell motility, proliferation, and cellular trafficking, highlighting the importance of this regulation. This finding is consistent with previous studies both in magnitude and in functional diversity of affected genes [15]. Interestingly, our gene set enrichment analysis revealed an enrichment of RNA binding proteins in many of the significant gene sets (Fig. 8C**, Table S6**). On the one hand, this is surprising, because we show that LSSs are present in most genes, and NCL was identified as directly binding to ini-tRNA that is proposed to be a general component of the SOS mechanism that conveys the SOS components to the supraspliceosome [20]. Also, a previous analysis of the effect of heat shock on SOS revealed activation of latent splicing in hundreds of gene transcripts, reflecting a wide repertoire of functional groups [15]. On the other hand, this over-representation could be a reflection of the breadth of essential cellular functions performed by RNA binding proteins, and therefore, it is functionally critical that such genes are spliced properly. This is in line with recent observations that SS selection is tightly regulated in germ cells [68]. Additional explanations for the observed enrichment in RNA binding proteins could include interaction with tissue-specific splicing factors or recruitment of splicing factors having high affinity for specific motifs within the pre-mRNA of RNA binding proteins. Yet, other explanations cannot be excluded at this stage. Further experiments directed at deciphering the molecular interactions are required to resolve this issue.

### Proposed update of the SOS speculative model

**Figure 9.**
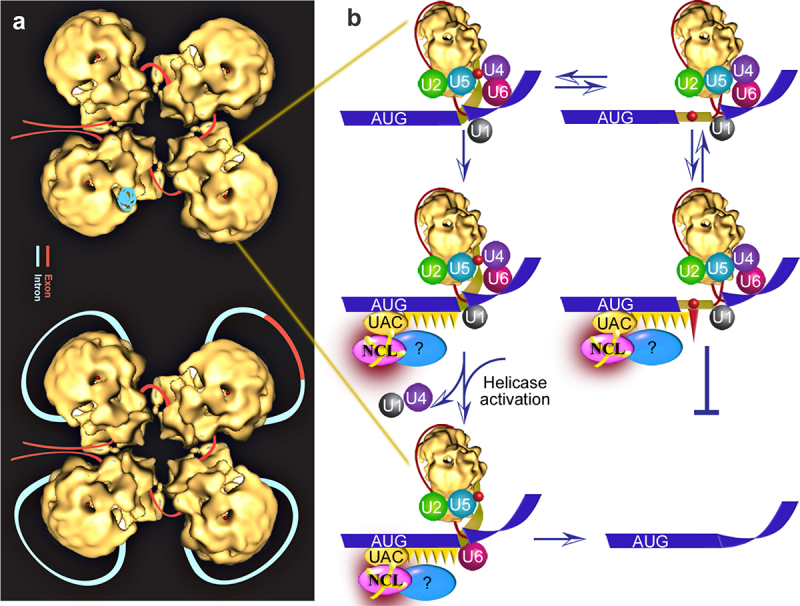
An updated speculative schematic model for the quality control function of SOS. (*A*) The supraspliceosome model [39,43,69,83,84]. Exon, red; intron, light blue. (Top) The folded pre-mRNA that is not being processed is protected within the cavities of the native spliceosome. (Bottom) When a staining protocol that allows visualization of nucleic acids was used, RNA strands and loops were seen emanating from the supraspliceosomes [85]. The RNA kept in the cavity likely unfolded and looped-out under these conditions. In the looped-out scheme an alternative exon is depicted in the upper right corner. (*B*) Zoom into one spliceosome. Left scheme, splicing at the authentic 5**′**SS; right scheme, splicing at the latent 5**′**SS. Blue stripes, exons; red line, intron; yellow narrow stripe, latent exon; red circle, in-frame STOP codon; circles, U snRNPs; Orange ellipse (UAC), initiator-tRNA; purple ellipse, NCL directly bound with ini-tRNA; and blue ellipse, additional associated components; Orange triangles, hypothesized triplet-binding proteins; red triangle, STOP-codon-binding protein. Updated from ref [20].

The proposed SOS working model [20] includes the role of ini-tRNA as an SOS factor. This invokes, however, a highly speculative sense triplet-recognition mechanism that can be interrupted by STOP codon-binding proteins. In this study, we identified NCL as a potential novel SOS factor by demonstrating that NCL is directly associated with ini-tRNA in the nucleus, that NCL and ini-tRNA are associated with pre-mRNA, that the recovery of SOS by ini-tRNA complementation is NCL dependent, revealing the functional requirement of NCL in SOS, and that its downregulation activates latent splicing at hundreds of gene transcripts. Using these results, we have updated our working model to include SOS as a quality control mechanism within the supraspliceosome that acts before splicing (Fig. 9). The supraspliceosome is composed of four native spliceosomes, which are connected by the pre-mRNA [27,39,69]. According to this model, first, splice site combinations are selected through the combinatorial interplay of positive and negative regulatory signals present in the pre-mRNA, which are recognized by trans-acting factors, resulting in the assembly of a supraspliceosome on each pre-mRNA (Fig. 9A). Second, SOS endorses the right combinations of splice junctions (Fig. 9B). The details of NCL’s role in SOS require further study.

We proposed that the SOS mechanism is based on three elements. First, the AUG sequence is recognized by the complementary anticodon (UAC) of the ini-tRNA, which is in a complex with auxiliary proteins [20]. NCL can now be added in the model as a protein directly binding ini-tRNA, likely along with additional proteins. This step helps to establish a register for the recognition of the reading frame. The second SOS element involves the cooperative polymerization of protein(s) that bind triplets of nucleotides and in the absence of a PTC it reaches the selected 5’SS; this step is the quality control ‘confirming’ step of SS selection that triggers the remodelling of the spliceosome to its functional state (Fig. 9B, left). The final SOS element is suppression of splicing in the presence of a PTC, perhaps through a competing interaction with a STOP-codon-binding protein (e.g. a release factor-like protein). The unproductive complex may undergo a conformational change and revert to the productive splicing complex involving the authentic 5’SS, as indicated by the double arrows (Fig. 9B, right). In cases where the quality control mechanism fails, for example in cases of stress, downstream mechanism/s (e.g. NMD in the cytoplasm) may engage to safeguard the robust control of the system.

### Conclusions

Our study identified NCL, a highly abundant and conserved protein, with multiple cellular functions involved in cancer, as a potential novel regulator of splice site selection and a component of the SOS mechanism that is proposed to protect cells from latent splicing that would generate transcripts with PTCs. We also identified novel splicing targets of NCL involved in multiple important cellular pathways and cell metabolism functions.

## Materials and methods

### Plasmids

CAD-Mut31 (first ATG mutated to ACG) minigene construct (Syrian hamster CAD), and the plasmid carrying mutant initiator tRNA:CTA-to-CGT were previously described [20].

### Model system

We chose the *Xenopus laevis* (*Xenopus*) oocyte system for our experiments because SOS appears to be evolutionarily conserved [22], and thus the high protein concentration in *Xenopus* oocyte nuclei could be used to identify the proteins interacting with ini-tRNA in the nucleus. Furthermore, this system has been successfully used as a model system to study numerous biological processes, including splicing [70,71], and we previously used this system to show that injected ini-tRNA, which functions in SOS, is uncharged with an amino acid [20].

### In vitro *transcription of ini-tRNA.*

We generated a DNA template using pTRM, a plasmid carrying the WT ini-tRNA gene [72] and specific primers in a PCR reaction according to the manufacturer’s instructions, and as detailed in Supplementary Methods. The RNA was purified using the miRNeasy kit (217,004 Qiagen), according to the manufacturer’s instructions, and analysed on a denaturing gel.

### In vitro *transcription of elongator-tRNA.*

We annealed 1 mM of a primer having the elongator ini-tRNA antiparallel sequence to 1 mM T7 primer (90°C for 3 minutes), and cooled it to room temperature (RT; annealing buffer: 10 mM Tris-HCl pH 7.5, 50 mM NaCl). Then, we followed the transcription procedure explained above.

### *Injection of ini-tRNA into the nuclei of* Xenopus *oocytes*

We extracted *Xenopus* oocytes as previously described [73,74], and as detailed in Supplementary Methods. A day after oocyte extraction, 10 fmol (20 nl) of ^32^P ini-tRNA was injected into the oocyte’s nucleus (Picospritzer, PLI-100; Medical Systems Corp.). After 30 minutes, nuclei were extracted manually, and nuclei were gently squeezed out into GV buffer (5 mM HEPES, 17 mM NaCl, 83 mM KCl). Each nucleus was gently washed and transferred to a new plate [75]. Radioactivity of nuclei was measured (Perkin-Elmer Tri-Carbs 2900 TR Liquid Scintillation), and only nuclei having over half of the injected ini-tRNA were further assayed. Nuclei were irradiated at 4°C with an energy of 0.8 Joule (or 0.5 Joule as indicated) [Ultra-Lum UV Cross-Linker (UVC 515)]. Nuclei were digested by RNase A [1.75X10^−3^ RNase A units (Sigma R-6513) per 50 µl sample volume] and run on 8.7% SDS PAGE (1 nucleus/well).

### In vitro *binding of ini-tRNA to nuclear extract of Xenopus oocytes.*

We incubated nuclear extract (one nucleus per reaction, as described above) with increasing quantities (0.5–2 picomol) of biotinylated ini-tRNA for 10 minutes and irradiated them with UV light (as described above).

### Affinity purification of biotinylated ini-tRNA and its crosslinked factors

We affinity purified ini-tRNA with crosslinked components using 5 µl (or else as indicated) C1 streptavidin magnetic beads (Invitrogen 65,001) in B&W buffer according to the manufacturer’s protocol. The pellet was resuspended in SDS loading buffer, boiled at 90°C for 5 minutes, and run on an 8.7% SDS PAGE.

### Preparation of purified supraspliceosomes

Supraspliceosomes were prepared from nuclear supernatants enriched in supraspliceosomes as previously described [38,43]. Briefly, nuclear supernatants were prepared from purified cell nuclei by microsonication of the nuclei and precipitation of the chromatin. The nuclear supernatant was fractionated on 10–45% (vol/vol) glycerol gradients, at 4°C in an SW41 rotor run at 41 krpm for 90 min [or an equivalent ω^2^t = 2500 (ω is in krpm; t is in hr)]. The gradients were calibrated using the tobacco mosaic virus as a 200S sedimentation marker. Supraspliceosome peak fractions were confirmed by Western blot, RT-PCR and by electron microscopy visualization. To prepare purified supraspliceosomes as previously described [38], fractions corresponding to the 200S supraspliceosome peak fractions were combined, dialysed, concentrated and rerun on a second 10–45% glycerol gradient, under equivalent conditions of the first run, and the purified supraspliceosomes were analysed.

### SMN clones

We have used two HeLa stable cell lines expressing SMN1 minigenes: one with a PP7 tag at the 5ʹUTR; and one lacking the tag, both prepared by Dr. H. Kotzer-Nevo [42]. Both clones express SMN1 minigene having exons 6, 7, 8 and IVS 6 and 7 cloned in pCDNA3. The plasmid was generated from the pCI-SMN2 plasmid [76], (kindly provided by Dr. C.L. Lorson, University of Missouri, through Dr. S. Stamm, University of Kentucky) by a C to T mutation at position 6 of exon 7. The plasmid pCDNA-SMN1-PP75ʹUTR was generated by inserting the PP7 tag to the XhoI site at the 5ʹUTR of SMN1. The two plasmids pCDNA-SMN1-PP75ʹUTR and pCDNA-SMN1 were each stably transfected into HeLa cells to generate stable cell lines as described [34]. The resulting cell-lines were analysed for the presence of the constructs by RNA extraction followed by RT-PCR and sequencing [42].

### Isolation of ZZTEVPPCP

The plasmid pET28-ZZTEVPP7CP, for expressing the ZZTEVPP7CP protein was kindly provided by Dr. K. Collins (University of California at Berkeley, Berkeley, CA, USA). The expression and purification of the protein was performed as described [41].

### Affinity purification of PP7-tagged splicing complexes

Nuclear supernatants enriched in supraspliceosomes were prepared as previously described [38,43] (see also above). The affinity purification was performed as described [41] with some changes as previously described [34], and without using the tobramycin step. All steps were conducted at 4°C, with mild agitation. To 500 μl of the nuclear supernatants, originated from ~0.5–1*10^8^ cells, 1 μg of ZZTEVPP7CP protein was added in a solution of: 10% (v/v) glycerol, 0.1% (v/v) NP-40, 0.5 mM phenylmethylsulfonyl fluoride (PMSF), final concentration; and incubated for 90 min with rotation. Next, 250 μg of IgG agarose beads (Sigma Aldrich) pre-washed with binding buffer [10% (v/v) glycerol, 10 mM Tris pH 8, 2 mM MgCl_2_, 100 mM NaCl, 1 mM DL-Dithiothreitol (DTT), 0.1% (v/v) NP-40, 0.5 mM PMSF and 2 mM vanadyl ribonucleoside (VR)] were added, followed by additional rotation for 90 min. Three washing steps with binding buffer and another one with TEV buffer (10% (v/v) glycerol, 10 mM Tris pH 8, 2 mM MgCl_2_, 100 mM NaCl and 1 mM DTT) were performed for 10 min each. Elution of the bound material from the beads was performed by incubating the beads overnight with 1 μg of TEV protease in 50 μl TEV buffer. The supernatant was then transferred to a new tube. Samples were taken either for protein analysis by WB and for RNA analysis by RT-PCR.

### Transfection experiments

Human 293 T cells were grown to 20–40% confluency in tissue culture plates and transiently cotransfected with the appropriate constructs as described [20]. Cells grown in 6-well plates were either treated or not with si-RNA (see below). 48 h after si-transfection, cells were transfected with plasmids expressing CAD Mut31, and mutant ini-tRNAs at 1 µg and 5 µg per 5 × 10^6^ cells, respectively using PEI-max reagent. Cells were harvested at 24 h (CAD) post transfection and total cellular RNA was extracted using the RNeasy kit (Cat #74,104, Qiagen), according to the manufacturer’s instructions (for the knockdown experiment see below).

#### RNA analysis

RNA extraction was performed as described [34]. Samples (up to 250 μl) in 10% (v/v) glycerol, 10 mM Tris pH 8, 100 mM NaCl, 2 mM MgCl_2_ and 2 mM VR were mixed with 75 μl of extraction buffer (50 mM Tris pH 7.5, 150 mM NaCl) and 25 μl of 10% (w/v) SDS. The RNA was recovered by extraction with phenol and precipitation in ethanol. The RNA was treated with DNase I (50 U/ml; Promega). cDNA was synthesized from up to 3 µg of RNA, using dT15 primer. Reverse transcription of ini-tRNA was done using TGIRT-III Enzyme (InGex; 5,073,018) as described [77], using the RNA-DNA double-strand primers with T overhang described in Supplementary Methods, with the kind advise and help of the laboratory of Dr. Y. Pilpel (Weizmann Institute).

#### RT-PCR analysis

RT-PCR was performed on RNA extracted from the NCLsi and CONTsi treated cells and from splicing complexes as described [15], and detailed in Supplementary Methods, using the detailed sets of primers (see Supplementary Methods). Each experiment was repeated at least 3 times.

Please note that the two SMN1 primer pairs were designed to target specifically the SMN1 minigene pre-mRNA, and primer pair F1R of SMN1 amplify two pre-mRNA isoforms. The CAD primers were designed to target specifically the CAD minigene. The identity of all PCR products was confirmed by sequencing.

#### Western blot (WB)

We ran our samples on 8.7% and 12% SDS PAGE and transferred them to a nitrocellulose membrane as described before [34]. We performed WB analyses using anti-NCL antibodies (C23, cat # Sc-13,057, and sc-8031 Santa Cruz; ABIN183989 by antibodies-online GmbH, and ZN004 mouse monoclonal antibody, cat# 39–6400 ThermoFisher Scientific) and visualized our product with horseradish peroxidase-conjugated to affinity-purified goat anti-rabbit IgG and goat anti-mouse (1:5000 dilution; H + L; Jackson ImmunoResearch); anti-hnRNP G (kindly provided by Prof. Stefan Stamm, University of Kentucky, Lexington); anti – YWHAQ (Thermo Scientific) visualized with horseradish peroxidase-conjugated to affinity-pure goat anti-rabbit IgG (H + L; Jackson Immunoreaserch, 1:5000), and with anti-Sm, as described [34].

#### Mass spectrometry analyses

We incubated 60 *Xenopus* oocyte nuclei with 120 picomol ini-tRNA (assay samples were prepared with biotinylated UTP and control samples were prepared with non-biotinylated UTP). All samples were affinity purified using 150 µl C1 streptavidin magnetic beads (as described above) and then resuspended in 100 µl 10 mM Tris buffer pH 7.5. We used in-solution, on-bead, tryptic digestion to prepare samples, as detailed in Supplementary Methods. Liquid Chromatography and Mass Spectrometry analyses are detailed in Supplementary Methods.

#### Knockdown of NCL

We transfected siRNA targeted to NCL (L-003854-00-0005, ON-TARGETplus Human NCL (4691) siRNA – SMARTpool, Dharmacon), and non-targeting siRNA as control (D-001810-01-05 ON-TARGETplus Non-targeting siRNA #1, Dharmacon) into HEK 293 cells with TransIT-X2 (mc-MIR-6003, Mirus) according to the manufacturer’s instructions with some modifications. Cells grown in 6-well plates were transfected with 45 nM siRNA by using TransIT-X2 transfection reagent. After 24 h, medium was changed, and cells were transfected again, as before. After 48 h, total proteins and RNA were extracted (RNeasy Qiagen 74,104) according to the manufacturer’s instructions and analysed. For the knockdown of NCL in cells transfected with siRNA, and then transfected with CAD-Mut31 and the complementary mutant ini-tRNA construct, see above. For the NCL knockdown experiments with 22.5, and 45 nm siRNA, RNA and proteins were extracted after 24 hr.

#### FACS analysis

HEK 293 cells grown in 6-well plates were treated with siRNA for 48 hr, as for the RNA-seq experiment (45 nM siRNA, and after 24 h and medium change, transfected again, as before), using as controls untreated cells and cells transfected with non-targeting siRNA, as above. We also repeated the experiment using 25 nM siRNA. For cell cycle analysis, cells were collected after 48 hr of siRNA treatment, washed once with cold PBS, fixed in cold ethanol for 1 h, centrifuged, and resuspended in 0.4 ml of PBS, incubated with RNase A (10 mg/ml) for 15 min at 37°C, and then propidium iodide (0.5 mg/ml) was added. The cells were then subjected to flow cytometry analysis on Amnis CellStream Flow Cytometer (Luminex, Austin, USA), and analysed using CellStream Analysis Software.

#### RNA library preparation and sequencing

Using the TruSeq® Stranded mRNA Sample Preparation kit (Illumina), we prepared an RNA library from four samples [1]:1-NCL-siRNA (1-NCLsi) [2],2-si-CONTROL (2-CONTsi) [3],7-NCL-siRNA (7-NCLsi), and [4]8-si-CONTROL (8-CONTsi). Briefly, polyA fraction (mRNA) was purified from 500 ng of total RNA for each sample, fragmented, and used to generate double stranded cDNA. We then performed end repair, A base addition, adapter ligation, and PCR amplification of the samples. We evaluated the libraries with Qubit and TapeStation. Sequencing libraries were constructed with barcodes to allow multiplexing of four samples on four sequencing lanes, which were then sequenced with the Illumina HiSeq 2500 V4 instrument. More than 234 million paired-end, 126-bp reads were generated in total for each sample (**Table S3**).

#### Identification of latent 5’SSs and alternative donor SSs

We compiled a comprehensive list of potential latent 5ʹ splice sites (LSSs) in the human genome by investigating all GT dinucleotides located in intronic regions of multi-exon coding transcripts. Upon passing a MaxEntScan score threshold of 0, the main criterion qualifying a GT dinucleotide as an LSS is the introduction of at least one in-frame STOP codon upstream (see Supplementary Methods for detailed information). Other alternative donor splice sites, such as adSS_3n_ (extend coding exons by lengths that are a multiple of three) and adSS_fs_ (extend coding exons by lengths that are not multiple of three), were selected only among GT dinucleotides that do not introduce upstream in-frame STOP codons.

#### Analysis of RNA-seq data

We performed read alignment with the STAR (v2.3.1) aligner [78] against the hg19 (GRCh37) assembly of the human genome (for further details see Supplementary Methods). We used the QoRTs package [79] to quantify reads mapped to exon junctions and genes included in the Gencode v19 annotation track. Differential gene expression analysis was performed with the DESeq2 package [80] in R (v3.2.2) with two case samples (1-NCLsi and 7-NCLsi) and two controls (2-CONTsi, 8-CONTsi).

#### Quantification of increased LSS usage

To compare LSS usage between samples, we used the counts of reads mapped to the junction formed by the LSS and the downstream acceptor SS normalized by the counts of reads mapped to the corresponding canonical junction (i.e. # split-reads_LSS_/# split-reads_5’SS_). As candidates for activation of latent splicing we considered those LSSs for which we observed at least one NCLsi replicate with at least four reads supporting the LSS and a fold increase ≥1.5 in normalized LSS usage in the NCLsi compared to the CONTsi sample. Additional conditions imposed to minimize the number of false positives are described in the Supplementary Methods.

#### Analysis of expression profiles

To further verify that the observed increase in LSS usage corresponds to the extension of the upstream exon into the intronic region we built expression profiles by quantifying reads mapped throughout the exon extension region. We compared normalized NCLsi and CONTsi profiles and retained cases for which both replicates were consistent with the extension of the upstream exon using two main criteria: *i*) reads were present throughout the exon extension region in the NCLsi sample (but they can be absent from the CONTsi sample), and *ii*) normalized expression levels were consistently higher in NCLsi compared to CONTsi samples throughout the exon extension region. For further details, see Supplementary Methods.

#### Gene set analysis

Overall, LSSs were assigned to a total of 17,975 Entrez gene IDs, with only 7,287 LSSs (0.5%) remaining unassigned. Enrichment of various biological properties among genes with activated LSSs was evaluated with the hypergeometric test against curated gene sets from the Molecular Signatures Database (Version 6.2; MSigDB) [81,82]. Additional details are available in the Supplementary Methods.

### Data access

The sequencing data for this study have been submitted to the NCBI Gene Expression Omnibus (GEO) under the accession number GSE142836.

## Supplementary Material

Supplemental Material
