# Supplementary material for: A novel role for nucleolin in splice site selection
Source: RNA Biol. 2022 Feb 27;19(1):333–52. doi: 10.1080/15476286.2021.2020455 (PMC8890436; doi:10.1080/15476286.2021.2020455)
Supplement: Supplemental Material [file KRNB_A_2020455_SM1019.zip › Supplementary information/Shefer_et_al_RNA_Biol_Supplementary_Information.docx.pdf]

**RNA Biol.** ***In press*** KRNB2020455
Manuscript DOI: 10.1080/15476286.2021.2020455

# A Novel Role for Nucleolin in Splice Site Selection

Kinneret Shefer[1,#], Ayub Boulos[1,#], Valer Gotea[2,#], Maram Arafat[1,#], Yair Ben Chaim[3], Aya Muharram[1], Sara Isaac[4], Amir Eden[4], Joseph Sperling[5], Laura Elnitski[2,*] and Ruth Sperling[1,*]

## Supplementary Information

## Supplementary Figure

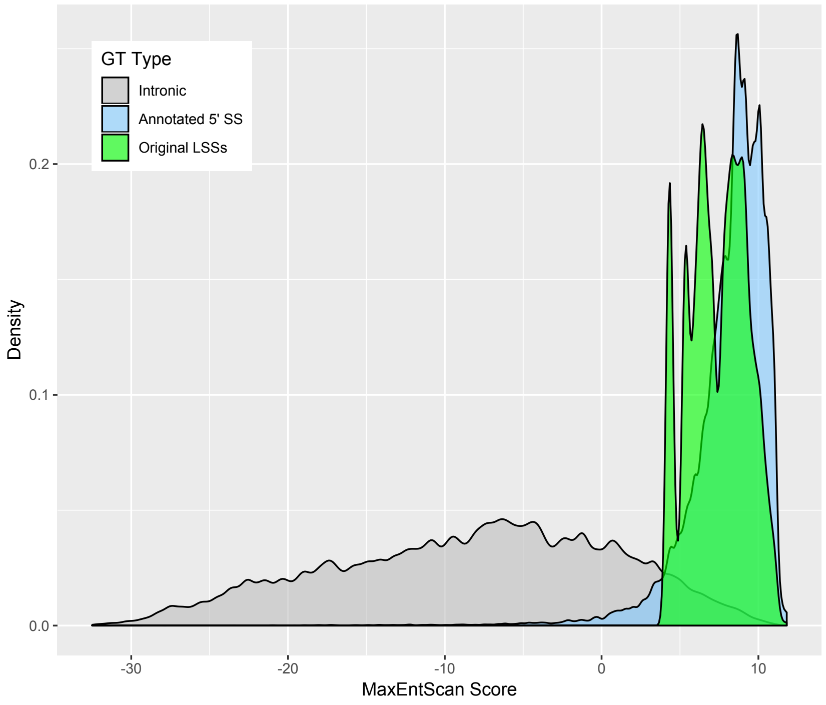


**Figure S1.** Distribution of MaxEntScan scores for GT dinucleotides in the human genome. The "Intronic" set includes 7762681 GTs located in within-CDS introns. They are limited to those GTs that would extend the upstream exon by at most 1000 nt and would allow a downstream intron of at least 20 nt. The "annotated 5' SS" set includes 189037 GTs annotated as donor (5') SSs in NCBI RefSeq, UCSC knownGene, or Gencode (v19) transcripts. Only within-CDS SSs were considered, and SSs with non-canonical consensus dinucleotides were excluded. The "Original LSSs" include 42305 GTs previously identified as LSSs by Nevo *et al*. (15). Their scores are based on the hg18 assembly of the human genome, whereas the other two sets are based on the data from the hg19 assembly.

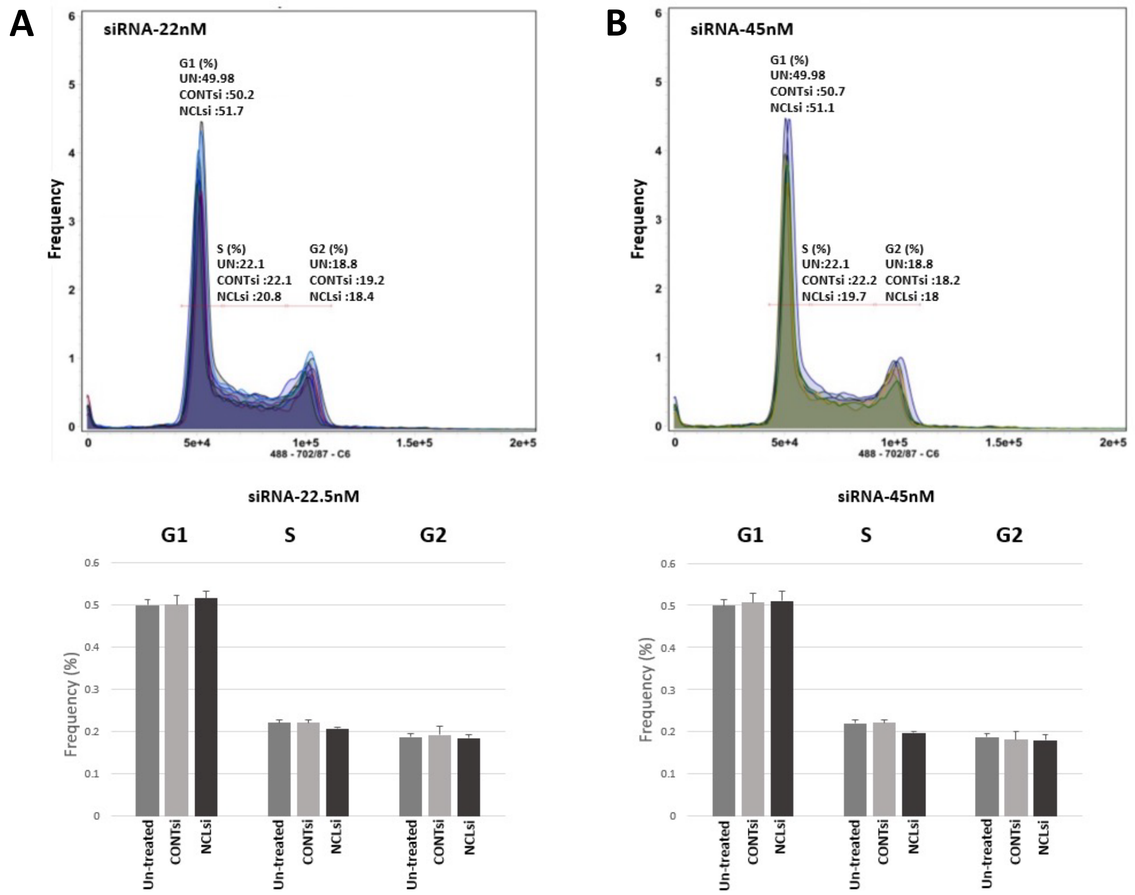


**Figure S2. Analysis of cell cycle in cells treated with siRNA directed against NCL.** HEK 293 cells were treated by siRNA against NCL (NCLsi). As controls, we used non-targeting siRNA (CONTsi), and untreated cells. Transfection of siRNA was at 22.5 nM (A) and 45 nM (B) for 48 hr, as described in Materials and Methods, and the cells were then analysed by FACS. The experiment was performed in three biological repeats of each sample. Densitometry analysis and histograms show the percentage of cell cycle distribution in G1, S and G2/M phases.

## Supplementary Methods

***In vitro* Transcription of ini-tRNA.** We generated a DNA template using pTRM, a plasmid carrying the WT ini-tRNA gene (72), Q5 DNA polymerase (M0491 NEB), and specific primers in a PCR reaction according to the manufacturer's instructions. *In vitro* transcription was carried out at 37ºC for 1 hour in a reaction containing 1.5 µg DNA template, 0.5 mM NTPs, 1.5 µci $\alpha$–$^{32}$P UTP (1.8 µl BLU007H250UC, Perkin-Elmer), X1 transcription buffer, and 30 units T7 RNA polymerase (Ambion 2718). To generate biotinylated ini-tRNA, we added 0.02 mM biotin UTP to the transcription reaction. To produce cold biotinylated ini-tRNA, we added 0.06 mM cold UTP and 0.02 mM biotinylated UTP to the transcription reaction. After DNase digestion [10 U DNase (Fermentase M610A), 37°C, 20'], the RNA was purified using the miRNeasy kit (217004 Qiagen), according to the manufacturer's instructions, and analyzed on a denaturing gel.

**Injection of ini-tRNA into the Nuclei of *Xenopus* Oocytes**. We extracted *Xenopus* oocytes as previously described (73, 74). Briefly, *Xenopus* oocytes were isolated and incubated in NDE96 solution composed of ND96 (96 mM NaCl, 2 mM KCl, 1 mM $CaCl_2$, 1 mM $MgCl_2$, 5 mM Hepes-NaOH, pH 7.5), with the addition of 2.5 mM sodium pyruvate, 100 units/ml penicillin, and 100 µg/ml streptomycin. A day after oocyte

extraction, 10 fmol (20 nl) of $^{32}$P ini-tRNA was injected into the oocyte's nucleus (Picospritzer, PLI-100; Medical Systems Corp.). After 30 minutes, nuclei were extracted manually using a small needle to pierce the oocyte membrane, and the nuclei were gently squeezed out into GV buffer (5 mM HEPES, 17 mM NaCl, 83 mM KCl). Each nucleus was gently washed and transferred to a new plate (75). Radioactivity of nuclei was measured (Perkin-Elmer Tri-Carbs 2900 TR Liquid Scintillation), and only nuclei having over half of the injected ini-tRNA were further assayed. Nuclei were irradiated at 4ºC with an energy of 0.8 Joule (or 0.5 Joule as indicated) [Ultra-Lum UV Cross-Linker (UVC 515)]. Nuclei were digested by RNase A [$1.75X10^{-3}$ RNase A units (Sigma R-6513) per 50 µl sample volume] and run on 8.7% SDS PAGE (1 nucleus/well).

**RT-PCR Analysis**. RT-PCR was performed on RNA extracted from the NCLsi and CONTsi treated cells and from spliceosome complexes as described (15), using the detailed sets of primers (see Primer List). The identity of all PCR products was confirmed by sequencing. Each experiment was repeated at least 3 times. The relative abundance was quantified in view of the intensity of GAPDH or actin used as a control. Reverse transcription of ini-tRNA was done using TGIRT-III Enzyme (InGex; 5073018) as described (77), using the RNA-DNA double strand primers with T overhang described in Supplementary Methods, with the kind help of the laboratory of Prof. Y. Pilpel (Weizmann Institute). Specifically, the RNA sample with the above RNA-DNA double strand primers and TGIRT-III Enzyme were incubated at room temp for 30 min. Next dNTPs were added and the reaction was carried out at 60°C for 60 min. PCR for ini-tRNA was performed with the detailed list of primers, with first denaturation of 15 min.

**Mass Spectrometry Analyses**

**Samples Preparation.** We incubated 60 *Xenopus* oocyte nuclei with 120 picomol ini-tRNA (assay samples were prepared with biotinylated UTP and control samples were prepared with non-biotinylated UTP). All samples were affinity purified using 150 µl C1 streptavidin magnetic beads (as described above) and then resuspended in 100 µl 10 mM Tris buffer pH 7.5.

We used in-solution, on-bead, tryptic digestion to prepare samples. We added 8 M urea in 0.1 M Tris (pH 7.9) to the beads and incubated them (15 min at RT). Proteins were reduced by incubation with dithiothreitol (5 mM; Sigma; 60 min at RT) and alkylated with 10 mM iodoacetamide (Sigma) (30 min at RT in the dark). We diluted the urea solution to 2 M with 50 mM ammonium bicarbonate, added 250 ng trypsin (Promega; Madison, WI, USA), and incubated the reaction overnight at 37°C. We then added 100 ng trypsin for 4 hr at 37°C and stopped the digestion with 1% trifluroacetic acid (1% final concentration). Peptides were desalted using Oasis HLB µElution format (Waters, Milford, MA, USA), vacuum dried, and stored at -80˚C until further analysis.

**Liquid Chromatography.** We used ULC/MS grade solvents for all chromatographic steps. Each sample was loaded using split-less nano-Ultra Performance Liquid Chromatography (10 kpsi nanoAcquity; Waters, Milford, MA, USA). The mobile phase was: A) $H_2O$ + 0.1% formic acid and B) acetonitrile + 0.1% formic acid. Desalting of the samples was performed online using a reversed-phase Symmetry C18 trapping column (180 µm internal diameter, 20 mm length, 5 µm particle size; Waters). The peptides were then separated using a T3 HSS nano-column (75 µm internal diameter, 250 mm length, 1.8 µm particle size; Waters) at 0.35 µL/min. Peptides were eluted from the column into the mass spectrometer using the following gradient: 4% to 30%B in 30 min, 30% to 90%B in 10 min, maintained at 90% for 5 min, and then back to initial conditions.

**Mass Spectrometry.** We coupled the nanoUPLC online through a nanoESI emitter (10 µm tip; New Objective; Woburn, MA, USA) to a quadrupole orbitrap mass spectrometer (Q Exactive Plus, Thermo Scientific) using a FlexIon nanospray apparatus (Proxeon). We acquired data in data dependent acquisition (DDA) mode, using a Top20 method. MS1 resolution was set to 70,000 (at 400m/z), mass range of 300-1650m/z, AGC of 3e6 and maximum injection time was set to 20msec. MS2 resolution was set to 17,500, quadrupole isolation 1.7m/z, AGC of 1e6, dynamic exclusion of 60sec and maximum injection time of 60msec.

**Identification of Latent 5'SSs and Alternative Donor SSs.** The list of LSSs was obtained with custom Perl software designed to scan the intronic sequences of protein-coding loci corresponding to transcripts from NCBI RefSeq (September 18, 2016), UCSC knownGene (June 30, 2013), and GENCODE v19 (December 5, 2013) annotation tracks. For this purpose we use the hg19/GRCh37 assembly of the human genome. Five criteria were used to define GTs as LSSs: *i*) they were located in introns flanked by coding

exons, *ii*) they were no more than 1000 nucleotides downstream of the annotated 5'SS, and accommodating an intron of at least 20 nucleotides with the downstream 3'SS, *iii*) they scored higher than 0 on the MaxEntScan scale (44), *iv*) they were not previously annotated as the 5'SS of a separate alternative exon fully contained within the intron investigated, and *v*) the translation of the intronic region up to the LSS (i.e., the exon extension) introduced at least one premature termination codon (PTC). For LSSs contained in overlapping transcript isoforms, i.e., those with different reading frames and intron boundaries, analyses were performed separately for each isoform, and only unique LSS instances are reported for LSS counts. With the exception of the requirement for insertion of in-frame STOP codons, the same criteria were used for identifying other alternative donor splice sites ($adSS_{3n}$, $adSS_{fs}$). Cases of $adSS_{3n}$ that introduce new STOP codons that span the exon junction (a total of 805 cases) were considered as $adSS_{fs}$.

**Analysis of RNA-Seq Data.** For the genome indexing step of the STAR workflow, we included GENCODEv19 gene annotation information and information for all junctions involving LSSs and adSSs. For the alignment step, we set the "--alignSJDBoverhangMin" parameter to 2 to increase alignment sensitivity at junctions of interest, which included both previously annotated junctions and our lists of LSSs and adSSs. Overall, about 84% of reads from all four samples were uniquely mapped (**Table S3**) and used for further analyses.

**Quantification of Increased LSS Usage.** Split reads, i.e. those aligned over an intron-induced gap to exons flanking each side, can usually considered as proof of transcript undergoing the process of splicing. However, when considering cases with increased LSS usage (i.e. fold increase of at least 1.5 in the case over the control sample), we imposed additional conditions to minimize the rate of false positives due to technical artifacts. In addition to a minimum number of 4 reads required, these conditions included: *i*) the second replicate was required not to exhibit a decrease in LSS usage (i.e. increase in normalized LSS usage was require to be ≥1), *ii*) at least one split reads was required to support the LSS in the second NCLsi replicate, and *iii*) both CONTsi replicates were required to have at least 10 split reads that support the canonical junction to indicate that minimal expression can be detected for the canonical junction under normal conditions.

**Analysis of Expression Profiles.** In building the expression profiles for the exon extension regions we excluded all read pairs for which any part of either read mapped to an intronic region. In this way we minimize the impact of intronic transcriptional noise on evaluating the expression profiles, which is variable across the human genome. Additionally, in evaluating the expression profile, we allowed small deviations from the condition of consistent higher expression level in the NCLsi sample in order to accommodate situation in which reads not originating from the transcripts of interest (e.g. reads generated by intronic transcriptional noise that fully map to exonic regions, reads that originate from alternative isoforms) cannot be readily eliminated. Our pipeline allowed us to eliminate many cases inconsistent with activation of latent splicing at LSS but which showed an increase in LSS usage. Such cases included, but are not limited to, unannotated exons, intronic alternative transcription start sites, or specific amplification of split reads that can happen during construction of sequencing library.

**Gene Sets Analysis**. Assignment of LSS-containing transcripts to Entrez gene IDs was performed using six cross-reference datasets: "gene2refseq" and "gene2ensembl" from NCBI (ftp://ftp.ncbi.nlm.nih.gov/gene/DATA/, accessed on October 30, 2018), "knownToRefSeq and "knownToEnsembl" from UCSC (http://hgdownload.soe.ucsc.edu/goldenPath/hg19/database/, accessed on October 30, 2018), the GENCODE v19 annotation set, as well as a custom reference dataset downloaded through the BioMart interface (https://www.ensembl.org/biomart, accessed on October 30, 2018) containing: Ensembl gene ID, Ensembl transcript ID, NCBI RefSeq and UCSC transcript accession numbers, gene symbol, and NCBI Entrez gene ID. The sets of curate genes were obtained from version 6.2 of MSigDB, which contains 8 gene set collections. Each collection contains between 50 and 5917 individual gene sets (17,810 gene sets in total). Each gene set consists of between 5 and 2940 unique gene identifiers, provided as gene symbols and Entrez gene IDs. To correctly evaluate enrichment in biological properties for the genes with activated LSSs, all genes included in the analysis (i.e. genes from the MSigDB gene sets, as well as genes in the background set) were required to have evidence of *i*) at least one LSS with a MaxEntScan score above 0.05 and *ii*) support of at least 10 split reads in both CONTsi samples for the

canonical junction encompassing the suitable LSSs in order to match the genes that could be detected as having activated LSSs.

**Primer List**
Specific primers for **ini-tRNA** generation were designed:
Sense primer: 5'- TAATACGACTCACTATAAGCAG - 3',
and antisense primer: 5'- TGGTAGCAGAGGATGGTTTC- 3'

**elongator ini-tRNA** template primer:
5'TGGTGCCCCGTGTGAGGATCGAACTCACGACCTTCAGATTATGAGACTGACGCGCTACCTA CTGCGCTAACGAGGCTATAGTGAGTCGTATTA-3
T7 primer: 5'-TAATACGACTCACTATA-3'

Primer sets for the RT-PCR validation experiments:

**RAF1**
Sense primer 5'- TCTGGCATGTTGAGGGCTTTG -3'
Antisense primer 5'- ACTTTGGTGCTACAGTGCTCA - 3'
Annealing temp 57˚C

**ANK2**
Sense primer 5'- GAGCTTGTGTCCTCTACATG - 3'
Antisense primer 5'- TCCAGCTGTCCCCATTCTCA - 3'
Annealing temp 56˚C

**VRK2**
Sense primer 5'- GCAAAGCAAGCTACTTCCAT - 3'
Antisense primer 5'- AATTCAGTCAGACCAGATCC - 3'
Annealing temp 51.5˚C

**KIAA0169**
Sense primer 5'- TGTGGCACAGCCCCTATTCCA - 3'
Antisense primer 5'- AGACCAAAGGTTCCCAAGGTG - 3'
Annealing temp 58˚C

**COPA**
Sense primer 5'- CAGAAGCGGTAACATATCAGG - 3'
Antisense primer 5'- CGTGTTTGGCTAGTAGTGCT - 3'
Annealing temp 51˚C

**LARS**
Sense primer 5'- TTGCGGCAGAAAATAGACCTA - 3'
Antisense primer 5'- GTCACACAGAGCCACAACAC - 3
Annealing temp 55˚C

**HTT**
Sense primer 5'- CAGGTCGTCCTTGTGTTAGGA - 3'
Antisense primer 5 - CTTGCATGGTGGAGAGACGA - 3'
Annealing temp 57˚C

**TCERG1**
Sense primer 5'- GTCAGAAAACAATCTTTGGGGG - 3'
Antisense primer 5'- TGTGCAACTCCTTCTCCCAC - 3'
Annealing temp 55˚C

**HEATR1**
Sense primer 5'- CAATTGAGAAATGGTGCTAGTC - 3'
Antisense primer 5'- TGATGAATGATGGAGACGACCA - 3'
Annealing temp 51.5˚C

**GAPDH**
Sense primer 5′- CCAGCCGAGCCACATCGCTC - 3′

Antisense primer 5′ - TGAGCCCCAGCCTTCTCCAT - 3′
Annealing temp 60.0˚C

Primers for RT-PCR of

**SMN1**
Forward primer 1 (exon 7) 5'- CCTTACAGGGTTTTAGACA-3'
Forward primer 2 (IVS 7) 5'-GTTGGTTGTGTGGAAGA-3'
Reverse primer 5'-TTTAGTGGTGTCATTTAGTG-3'
Annealing temp 50.0˚C

**CAD minigene**
Pre-mRNA forward primer 5'- AGAGAGGAGTTTACGTTGGGAGG -3'
Annealing temp 65˚C
Authentic mRNA forward primer 5'- ACCAAACTCTTCGTGGAGGCC -3'
Annealing temp 65˚C
Latent mRNA forward primer 5'- TCCAGAGGCGGAGAGGC -3'
Annealing temp 68˚C
Reverse primer 5'- CAGGGAGCCGCACCAGTTTC -3'.

**Primers for RT-PCR of ini-tRNA**
**For cDNA**
Reverse transcription DNA 5'-CACGACGCTCTTCCGATCTT-3'
Reverse transcription RNA 5-rArGrArUrCrGrGrArArGrArGrCrGrUrCrGrUrG-3'

**For PCR**
Sense primer 5'-AGCAGAGTGGCGCAGCGGAA-3'
Reverse primer 5'-TGGTAGCAGAGGATGGTTTCGA-3'
Annealing temp 62.5˚C

**Supplementary Tables**

**Table S1.** List of peptides identified by mass spectrometry.

**Table S2.** List of latent splice sites (LSSs) identified in the human genome.

**Table S3.** Summary of reads obtained through the RNA-Seq experiment and mapped to each of the four samples.

**Table S4.** List of genes with differential expression in NCLsi compared to CONTsi samples.

**Table S5.** List of LSSs activated in NCLsi samples upon NCL knockdown.

**Table S6.** Gene sets over-represented among the genes with activated LSSs.

**Table S7.** List of LSSs activated in CONTsi samples.

**Table S8.** List of $adSS_{3n}$ splice sites identified in the human genome.

**Table S9.** List of $adSS_{3n}$ activated in NCLsi samples upon NCL knockdown.

**Table S10.** List of $adSS_{3n}$ activated in CONTsi samples.

**Table S11.** List of $adSS_{fs}$ splice sites identified in the human genome).

**Table S12.** List of $adSS_{fs}$ activated in NCLsi samples upon NCL knockdown.

**Table S13.** List of $adSS_{fs}$ activated in CONTsi samples.
